# MDM2 drives resistance to Osimertinib by contextually disrupting FBW7-mediated destruction of MCL-1 protein in EGFR mutant NSCLC

**DOI:** 10.1186/s13046-024-03220-7

**Published:** 2024-11-15

**Authors:** Jiaxin Liu, Lingyun Wei, Qing Miao, Sutong Zhan, Peilin Chen, Wei Liu, Liang Cao, Dong Wang, Hongbing Liu, Jie Yin, Yong Song, Mingxiang Ye, Tangfeng Lv

**Affiliations:** 1 Department of Respiratory Medicine, Nanjing Drum Tower Hospital, Affiliated Hospital of Medical School, Nanjing University, Nanjing, China; 2 Department of Thoracic Surgery, Jinling Hospital, Affiliated Hospital of Medical School, Nanjing University, Nanjing, China; 3 The guidance center for Military Psychology of PLA, The 960th Hospital of Joint Logistics Support Force of PLA, Jinan, China; 4 Department of Respiratory Medicine, Jinling Hospital, Affiliated Hospital of Medical School, Nanjing University, #305 East Zhongshan Road, Nanjing, 210002 China; 5 Liaoning Kanghui Biotechnology Co., Ltd, Shenyang, 110 167 China; 6 Department of Traditional Chinese Medicine, Tangdu Hospital, Air Force Medical University, Xi’an, CA 94404 China

**Keywords:** NSCLC, EGFR, Osimertinib resistance, MDM2, FBW7

## Abstract

**Background:**

Overcoming resistance to Osimertinib in epidermal growth factor receptor (EGFR) mutant non-small cell lung cancer (NSCLC) is clinically challenging because the underlying mechanisms are not fully understood. The murine double minute 2 (MDM2) has been extensively described as a tumor promotor in various malignancies, mainly through a negative regulatory machinery on the p53 tumor suppressor. However, the significance of MDM2 on the sensitivity to Osimertinib has not been described.

**Methods:**

Osimertinib resistant cells were generated by standard dose escalation strategy and individual resistant clones were isolated for MDM2 testing. The MDM2 and its mutant constructs (ΔPBD, ΔRING, C464A) were introduced into PC-9, HCC827 and H1975 cells and evaluated for the sensitivity to Osimertinib by MTT assay, colony formation, EdU assay and TUNEL assay. MDM2 expression in resistant cells was manipulated by pharmacological and molecular approaches, respectively. Proteins that were implicated in PI3K/Akt, MAPK/Erk and apoptosis signaling were measured by Western blot analysis. Candidate proteins that interacted with MDM2 were captured by immunoprecipitation and probed with indicated antibodies.

**Results:**

In comparison with parental PC-9 cells, the PC-9 OR resistant cells expressed high level of MDM2. Ectopic expression of MDM2 in PC-9, HCC827 and H1975 sensitive cells generated an Osimertinib resistant phenotype, regardless of p53 status. MDM2 promoted resistance to Osimertinib through a PI3K/Akt and MAPK/Erk-independent machinery, in contrast, MDM2 selectively stabilized MCL-1 protein to arrest Osimertinib-induced cancer cell apoptosis. Mechanistically, MDM2 acted as a E3 ligase to ubiquitinate FBW7, a well-established E3 ligase for MCL-1, at Lys412 residue, which resulted in FBW7 destruction and MCL-1 stabilization. Targeting MDM2 to augment MCL-1 protein breakdown overcame resistance to Osimertinib in vitro and in vivo. Finally, the clinical relevance of MDM2-FBW7-MCL-1 regulatory axis was validated in mouse xenograft tumor model and in NSCLC specimen.

**Conclusion:**

Overexpression of MDM2 is a novel resistant mechanism to Osimertinib in EGFR mutant NSCLC. MDM2 utilizes its E3 ligase activity to provoke FBW7 destruction and sequentially leads to MCL-1 stabilization. Cancer cells with aberrant MDM2 state are refractory to apoptosis induction and elicit a resistant phenotype to Osimertinib. Therefore, targeting MDM2 would be a feasible approach to overcome resistance to Osimertinib in EGFR mutant NSCLC.

**Supplementary Information:**

The online version contains supplementary material available at 10.1186/s13046-024-03220-7.

## Introduction

The gain-of-function alterations in epidermal growth factor receptor (EGFR) lead to its auto-phosphorylation and increased kinase activity, as a result, two major downstream signaling associated with cell survival and proliferation, PI3K/Akt and MAPK/Erk, are constitutively activated [1, 2]. Blockade of EGFR pathway by small molecule tyrosine kinase inhibitors (TKIs) has become the standard-of-care in patients with EGFR mutant non-small cell lung cancer (NSCLC) and defines a milestone of biomarker-based precision medicine [3]. The evolution of different generations of EGFR TKIs also provided increasing treatment options over the past two decades. Importantly, all the EGFR inhibitors have been demonstrated to be superior to platinum-doublet chemotherapy and substantially improved the quality of life in EGFR mutant patients [4, 5]. The third-generation EGFR TKIs Osimertinib is an irreversible EGFR inhibitor that selectively targets mutant EGFR and T790M resistance mutation. It significantly extends progression-free survival (PFS) in both previously untreated and heavily treated populations, and also in adjuvant settings [6–8]. Moreover, Osimertinib is a brain penetrable TKI and shows excellent central nervous system activity against brain and leptomeningeal metastatic diseases [9]. Thus, Osimertinib is licensed for first-line treatment of stage IV NSCLC with EGFR activating mutations and for patients who developed central nervous system metastasis and for whom progressed on first/second-generation EGFR TKIs due to T790M resistance mutation. Unfortunately, resistance to Osimertinib invariably emerges after years of treatment.

The most prominent mechanisms underlying Osimertinib treatment failure include: (1) A tertiary point mutation at the C797 residue that prevents covalent bond between EGFR and Osimertinib [10]. This C797S mutation occurs in 10–26% of Osimertinib-resistant cases in second line and 6–10% cases in first line settings [11], and the fourth-generation EGFR TKIs BLU-945 targeting C797S mutation is under early phase clinical evaluation. (2) Activation of bypass kinases, in particular c-Met amplification, which restores downstream survival outputs despite EGFR inhibition. The ongoing TATTON trail evaluating the efficacy of Osimertinib combined with Savolitinib, a potent c-Met inhibitor, in NSCLC patients who progressed on Osimertinib with c-Met-mediated machinery got encouraging results and represented a promising therapy to overcome resistance to Osimertinib [12]. (3) Histology transformation to small cell lung cancer (SCLC). This group of treatment tolerant patients mostly harbored *p53* and *Rb1* mutation with a response rate of 54% to etoposide/platinum chemotherapy [13]. Therefore, moving chemotherapy upfront to delay SCLC transformation is clinically considered. Although the current knowledge regarding resistance to Osimertinib is impressive, there is still a big gap in the understanding of resistance since the mechanism of resistance in more than 40% of cases has not been identified. Fortunately, clinicians are able to discover novel tumor promoting genes and resistance mutations with the broad implement of next generation sequencing (NGS). The NGS testing also yielded a panel of non-kinase co-occurring alterations that may not directly promote tumorigenesis, while they may have a remarkable impact on therapeutic outcomes. For example, co-mutation with p53 confers decreased sensitivity to EGFR TKIs and shortened PFS compared with patients without p53 mutation [14]. While the biological function of these non-kinase alterations is certainly underestimated, and one of these genetic events that attracts our attention is the aberration of murine double minute 2 (MDM2).

The *MDM2* proto-oncogene is frequently amplified or overexpressed in human malignancies, especially in liposarcoma and melanoma. According to a large scale screening of 10,587 biopsy samples from 7,121 patients with NSCLC, 6% of patients harbored *MDM2* amplification, with an enrichment in patients concurrently carrying EGFR mutation (8%) and ALK translocation (10%) [15]. Overexpression of MDM2 protein as a consequence of genetic amplification contributes to tumorigenesis, by which the best characterized model is MDM2 acting as a prime negative regulator for the tumor suppressor p53 [16]. The MDM2-p53 regulatory loop is therefore expected to be targetable in cancers with defined MDM2 aberration and p53 inactivation. While in early phase clinical trials evaluating the efficacy of small molecule inhibitors targeting MDM2, the inhibitors elicited limited single-agent activity. As such, MDM2 probably endowed tumor promoting potency independent of p53 [17].

In this study, we demonstrated that MDM2, a largely underestimated candidate for targeted therapy, as a novel resistance mechanism to Osimertinib in EGFR mutant NSCLC. The protein level of MDM2-FBW7-MCL-1 are inversely regulated in a coordinated manner in Osimertinib resistant cells. We found that MDM2 targets FBW7 for K48-linked polyubiquitination and proteolysis, which leads to defects in Osimertinib-induced cell apoptosis process. Notably, concurrent inhibition of EGFR and MDM2 efficiently triggered MCL-1 degradation and overcame Osimertinib resistance. Our study thus established a previously undefined apoptosis restriction cascade integrating E3 ligases and BCL-2 family proteins to determine the sensitivity to Osimertinib through sequentially targeting each other for degradation.

## Materials and methods

### Cell culture and reagents

The human NSCLC PC-9 (EGFR E746_A750 del) and H1975 (EGFR L858R/T790M) cells were purchased from ATCC and have been extensively described. HCC827 (EGFR E746_A750 del) cells were generously gifted by Jeffrey Engelman (Novartis Institutes for BioMedical Research). These cells were authorized by short tandem repeat analysis and tested for mycoplasma contamination before use. Cells were maintained in RPMI-1640 medium supplied with 10% fetal bovine serum (FBS) and 1% penicillin–streptomycin in 37 °C with an atmosphere of 5% CO_2_. The HEK293 cells were cultured with DMEM medium with 10% FBS and 1% penicillin–streptomycin.

Osimertinib and AZD5991 were purchased from Selleck Chemicals. Cycloheximide, MG132, MTT, puromycin and DAPI were purchase from Sigma-Aldrich. Matrigel was purchased from BD Bioscience. MX69 was purchased from MedChemExpress.

### Generation of Osimertinib resistant cells

Cells with acquired resistance were derived by treating parental PC-9 cells with increasing concentrations of Osimertinib starting at 20 nmol/L, followed by a stepwise dose escalation every 48 h up to 5 µmol/L until the emergence of resistant clones. Cells were washed and replenished with fresh drug every 48 h. Resistant cells were maintained in 1 µmol/L of Osimertinib.

### Western blot analysis and immunoprecipitation

Protein lysates were prepared with cell lysis buffer with protease and phosphatase inhibitors and protein concentrations were determined using BCA methods. Equal amount of protein samples was separated on SDS-PAGE gels and transferred to nitrocellulose membranes. The membranes were blocked in 5% non-fatty milk for 1 h at room temperature and incubated with indicated primary antibodies overnight at 4 °C, followed by corresponding horseradish peroxidase-conjugated IgG for additional 1 h. Protein bands were visualized by the chemiluminescence detection system (BioRad) and analyzed with ImageJ. Protein bands were quantified and normalized to GAPDH. The source of antibodies was listed in Table S1.

For immunoprecipitation assay, whole cell lysate (WCL) was incubated with specific antibodies or isotype IgG with gentle rotation overnight at 4 °C. The mixture was incubated with 50 µL of protein A/G sepharose beads (Santa Cruz) for additional 2 h before centrifugation. The deposition was mixed with equal volume of SDS buffer and boiled for 10 min. The supernatant was collected and loaded for electrophoresis.

### Plasmids generation and mutagenesis

HA-tagged FBW7, Myc-tagged FBW7, His-tagged Ub WT, Ub K48, and Ub K63 plasmids were preserved in our in-house plasmid bank as previously described [18]. Full length MDM2 cDNA with an N-terminal Flag tag was PCR amplified from pENTR221-MDM2 (Invitrogen) and inserted into pCMV3 vector. The Flag-MDM2 ΔRING, ΔPBD, ΔNLS, C464A and HA-FBW7 ΔQ and K412R mutants were generated using the Quick-Change Site-Directed Mutagenesis Kit (Stratagene). All constructs used in this study have been thoroughly sequenced.

To generate MDM2 lentivirus, the MDM2 cDNA or its mutants with N-terminal Flag tag was constructed into pCDH-puro vector. The pCDH-MDM2 construct was transfected into HEK293 cells together with packing plasmids (psPAX2 and pMD2.G) using Lipofectamine 3000 (Invitrogen). Forty-eight hours after transfection, the lentivirus particles containing supernatant was collected and then concentrated by superhigh speed centrifugation at 4 °C. Cells with stably expression of MDM2 or its mutant were obtained by infecting with indicated lentivirus and selecting with 2 µg/mL puromycin.

### Cycloheximide chase assay

After indicated treatment, cycloheximide (CHX) at final concentration of 25 µg/mL was added into cell culture medium. Cells were collected at different time intervals and prepared for Western blot analysis as previously described.

### MTT and EdU assays

Cells were seeded into 96-well plates at the density of 5000 cells per well. After indicated treatment, cells were incubated with 20 µL of MTT solution for additional 4 h. The formazan crystals were dissolved in DMSO and the plates were read in a microplate reader (BioRad, USA) at absorbance values at 490 nm.

For 5-Ethynyl-2’-deoxyuridine (EdU) labelling assay, cells were seeded on the coverslips in 6-well plates and incubated with or without 1 µmol/L Osimertinib for 24 h. EdU (RiboBio, China) was added to each well for additional 2 h, and the cells were fixed and permeabilized with 0.5% Triton X-100 at room temperature for 10 min. Cells were incubated with an Apollo^®^ reaction cocktail for 30 min and counter stained with Hoechst for 5 min. Cell proliferation was assessed with the proportion of EdU-positive nucleus under a fluorescence microscope (Olympus, Japan).

### Colony growth assay

Cells were seeded in 6-well plates at a density of 1000 cells per well. Appropriate drugs were added after 24 h. Cells were exposed to indicated drug or DMSO for 9–10 d, with medium and drug refreshed every 72 h. Cell colonies were fixed with 4% formaldehyde and stained with 0.5% crystal violet. Pictures of cell colonies were taken using a scanner.

### Subcutaneous xenograft tumor model

The NSCLC cells (5 × 10^5^) were suspended in 200 µL of diluted Matrigel and injected into the left flank of 4-weeks old nude mice. After the size of subcutaneous tumor reached 200 mm^3^, treatment was initiated. Mice were administrated with saline or inhibitors as indicated, including gastric gavage of Osimertinib (5 mg/kg), intraperitoneal injection of MX69 (20 mg/kg) and their combination. Tumor sizes were measured using a caliber twice a week and calculated using formula: length×width^2^/2. Animal care and experiments were performed in accordance with institutional ethical guidelines approved by the Institutional Animal Care and Use Committee (#2023JLHGZRDWLS-00032).

### Small interfere RNAs transfection

Small interfere RNAs (siRNAs) were synthesized by GenePharma (Shanghai) and dissolved in ddH_2_O. Cells were transfected with siRNAs at concentration of 10 nmol/L against MDM2, p53 or scrambled siRNAs control using Lipofectamine 3000 transfection reagent according to manufacturer instructions. Medium was refreshed 6 h after transfection, and cells were harvested for experiments 24 and 48 h post-transfection. Target sequences for siRNAs were available in Table S2.

### Patient selection for screening MDM2 data

We retrospectively collected a cohort of EGFR mutant NSCLC patients from 6 medical centers between August 2015 and March 2020 via chart review under an Institutional Review Board–approved protocol. The major inclusion criteria include EGFR activating mutation, treatment with Osimertinib without concurrent chemotherapy and eventually failed on Osimertinib, sufficient pre-treatment tissue available for NGS analysis, pre- and post-treatment radiographic images available for tumor measurements. Radiographs were reviewed by two experienced radiologists who were blinded to MDM2 results. RECIST methods were used to determine tumor burden and best response. Time-to-progression (TTP) was calculated as the time from the start of Osimertinib until documented progression by RECIST.

### Patient samples and immunohistochemistry

All patient tumor samples analyzed were obtained under Institutional Review Board Approved protocols with informed consent obtained from each patient under the guidance of Ethics Committee of Jinling Hospital, Affiliated Hospital of Medical School, Nanjing University (#2023DZGZR-030). Tissues were fixed in 4% paraformaldehyde overnight and embedded in paraffin. The xenograft tumor and patient samples were sectioned on slides with 4 μm thickness. The paraffin sections were deparaffinized in xylene and rehydrated in a graded alcohol series. Antigen retrieval was performed by boiling the slides in citrate buffer (pH = 6.1). Peroxidase activity was blocked using 0.3% H_2_O_2_. The slides were incubated with primary antibodies against MDM2 (1/50), FBW7 (1/50), MCL-1 (1/200) and cleaved caspase-3 (1/400) at 4 °C overnight. After 3 times washes in PBS, slides were incubated with horseradish peroxidase-conjugated secondary antibodies and DAB substrate (K3468, Dako). Two-steps Anti-Rabbit IgG Peroxidase Polymer Kit (PV-6001, ZSGB-BIO) and Anti-Mouse IgG Peroxidase Polymer Kit (PV-6002, ZSGB-BIO) were used according to the manufacturer’s instructions. Counterstain was performed with hematoxylin. The protein expression was scored based on the intensity of staining and the extent of staining. The staining area was scored as follows: 0, 0–5% of tissue stained positive; 1, 6–25% stained positive; 2, 26–50% stained positive; 3, 51–75% stained positive; and 4, > 75% positive cells. The staining density score index was designated as follows: 0, negative expression; 1, weakly positive; 2, moderate positive; 3, strongly positive. The IHC score was generated by multiplied the intensity score with density score from three different areas of the slides.

### Statistical analysis

Each experiment was performed at least in triplicate and all data were processed using GraphPad Prism Software. The experimental data were presented as mean ± standard deviation. Two-tailed unpaired or paired Student’s *t*-tests were performed for group comparisons. *P* value < 0.05 was viewed as significant.

## Results

### Identification of MDM2 amplification in Osimertinib resistant NSCLC patients

**Fig. 1:**
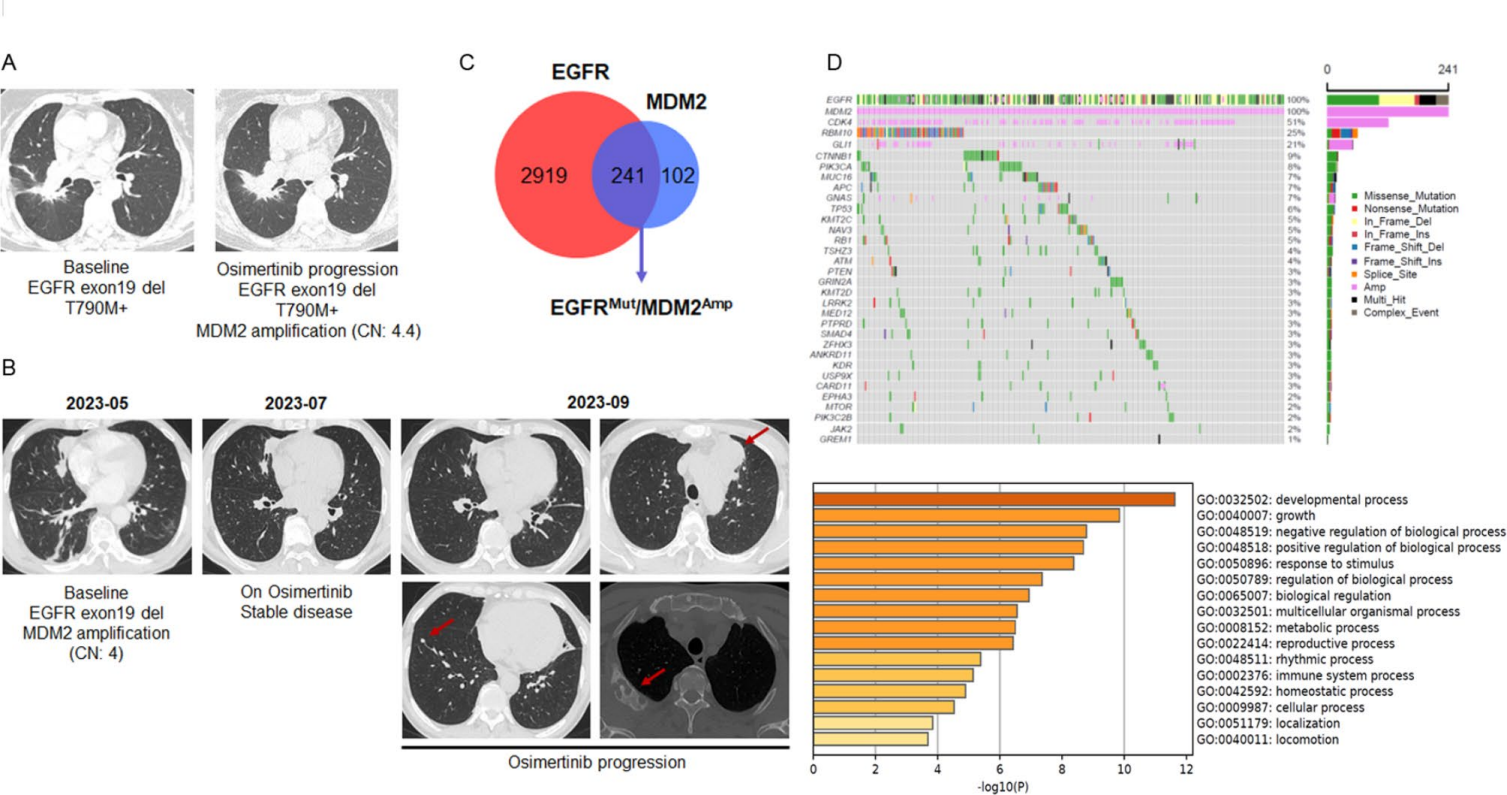
**Identification of MDM2 amplification in NSCLC with resistance to Osimertinib. (A)** The 61-years old female with stage IVB EGFR exon21 L858R mutant adenocarcinoma progressed on gefitinib due to T790M mutation. She began to receive Osimertinib as anti-cancer treatment in December 2016 and developed resistance in September 2019. The NGS testing of the repeated biopsy specimen showed the EGFR activating mutation and T790M mutation still existed after disease progression. However, MDM2 amplification was noted after Osimertinib resistance. **(B)** A 58-years old man with stage IVB lung adenocarcinoma referred to our institution in June 2023. The NGS result showed EGFR exon19 del and *de novo* MDM2 amplification. The patient was treated with first-line Osimertinib 80 mg per day and rapidly progressed on the treatment. Chest CT examination in September 2023 showed the emergence of multiple metastatic lesions indicated by red arrows. **(C)** Venn diagram showing the overlapping (purple part) of EGFR mutant NSCLC (red circle) and MDM2 amplified NSCLC (blue circle) according to NGS datasets consisting of 6,093 cases. **(D)** A patient cohort consisting of 241 cases of NSCLC concurrently harboring EGFR activating mutations and MDM2 amplification was analyzed. Tumor samples were arranged from left to right. Alterations of cooccurring genes were annotated for each sample according to the color panel below the image. The somatic mutation frequencies for each candidate gene were plotted on the right panel. Enrichment analysis and ranking of cooccurring genes in GO terms was performed using the online Metascape bioinformatic tool

We accidently noticed MDM2 amplification by NGS testing in a panel of NSCLC patients who failed on Osimertinib treatment. Notably, MDM2 amplification was detected as a solely additional genetic alteration after Osimertinib progression, without other defined resistance mutations. Although EGFR activating mutations still existed in the repeated biopsy samples, these MDM2 amplified tumors failed to respond to Osimertinib (Fig. 1A). Patients with *de novo* MDM2 amplification also rapidly progressed on Osimertinib (Fig. 1B). Thus, we speculated that amplification of MDM2 may contribute to resistance to Osimertinib in EGFR mutant NSCLC.

To validate this hypothesis, we first screened the MDM2 state in a total number of 6,093 cases of NSCLC in our NGS datasets. Among these patients, 3,160 cases (51.86%) harbored an EGFR activating mutation (Fig. 1C). Aberrance of MDM2 was found in 375 cases (6.16%), in which the frequency of SNP and amplification was 0.53% and 5.63%, respectively. Thus, the most prominent aberration of MDM2 in NSCLC is genetic amplification. Of noted, 241 patients concurrently harbored EGFR mutation and MDM2 amplification (Table 1). These patients tended to display distinct clinicopathological features, such as no smoking history, adenocarcinoma histology, and more advanced disease. Genetic profiling suggested an enrichment of tumor promoting gene sets (including CDK4, RBM10 and GLI1) in the EGFR^Mut^/MDM2^Amp^ cohort (Fig. 1D), while these candidate genes did not directly affect cell proliferation or cell death programs, we therefore concluded that MDM2 may not regulate sensitivity to Osimertinib at transcriptional level.

### Osimertinib resistant cells overexpressing MDM2

**Fig. 2:**
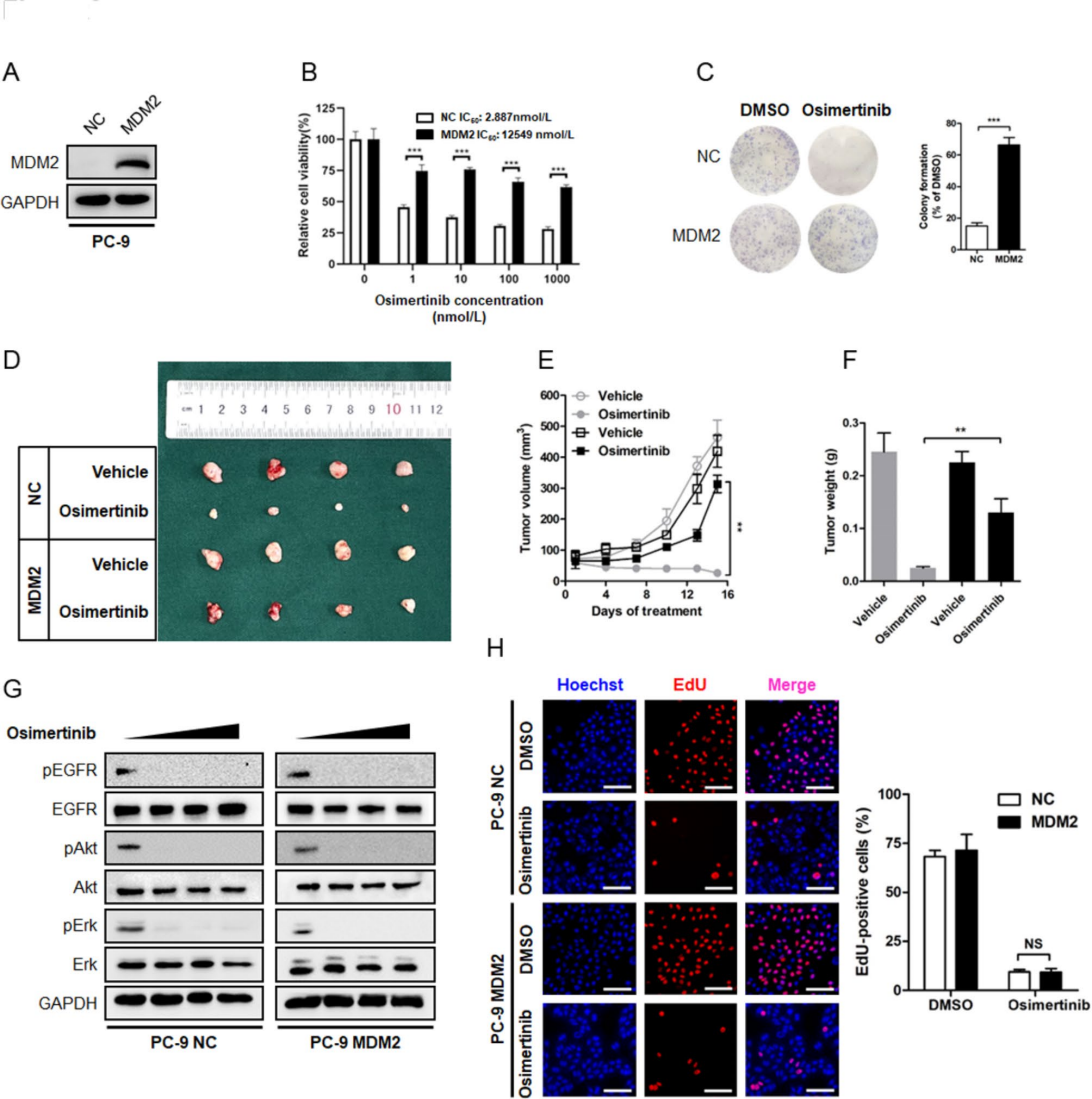
**Overexpression of MDM2 drives resistance to Osimertinib in EGFR mutant NSCLC. (A-C)** The parental PC-9 sensitive cells were engineered to stably express ectopic MDM2 or empty vector as a negative control (NC) and tested for the sensitivity to Osimertinib by MTT assay and colony formation assay, respectively. ***P < 0.001. **(D-F)** PC-9 cells stably expressing NC or MDM2 were seeded in nude mice and treated with Osimertinib (5 mg/kg) or equal amount of Vehicle. The tumor volume was routinely monitored. At the end of the experiment, the xenograft tumors were carefully removed, weighted and photographed. **P < 0.01 **(G)** PC-9 cells stably expressing NC or MDM2 were treated with increasing concentrations of Osimertinib (0, 0.1, 0.5, 1 µmol/L) for 6 h. After indicated treatment, whole cell lysate (WCL) was prepared following standard protocol and analyzed for the phosphorylation status of EGFR, Akt and Erk by immunoblotting. GAPDH was used as an equal loading control. **(H)** Representative images of EdU labelling assay. PC-9 NC and PC-9 MDM2 cells were treated with 1 µmol/L Osimertinib or equal amount of DMSO for 24 h. Cell proliferation was evaluated by EdU staining. Scale bar = 200 μm. NS: not significant

We modeled acquired resistance to Osimertinib by generating polyclonal acquired resistant cell pools on the basis of stepwise dose escalation over a period of 10 days followed by maintenance in 1 µmol/L of Osimertinib over 3 months. We isolated 7 resistant clones and tested MDM2 expression individually and found clone #3 (denoted by PC-9 OR cells) yielded significantly elevated *MDM2* transcripts when compared with parental PC-9 cells (Fig. S1). To characterize whether MDM2 is sufficient to drive resistance to Osimertinib, we overexpressed MDM2 in a panel of NSCLC cell lines harboring EGFR activating mutations. In comparison with NSCLC cells overexpressing empty vector as a negative control (NC), MDM2 readily induced a stable resistant phenotype to Osimertinib in PC-9 and HCC827 cells, as demonstrated by MTT cell viability assay and colony formation assay (Fig. 2A–C, Fig. S2A-S2C). Consistently, aberrant MDM2 expression also conferred resistance to Osimertinib in the T790M-positive H1975 cells (Fig. S2D-S2F). To explore whether MDM2 also promoted resistance to Osimertinib in vivo, we stably expressed MDM2 in PC-9 cells and seeded the cells in the flanks of nude mice. Treatment was initiated when the tumor nodules reached approximately 100 mm^3^. It was noted that PC-9 NC nodules were highly responsive to treatment, whereas the PC-9 MDM2 nodules continued to grow despite administration of Osimertinib (Fig. 2D). At the end of experiment, the average size and weight of xenograft tumor in PC-9 MDM2 group was 12-fold and 5-fold than that in PC-9 NC group, respectively (Fig. 2E F). Therefore, aberrant expression of MDM2 is sufficient to drive resistance to Osimertinib.

The PI3K/Akt and MAPK/Erk signaling has been extensively described as major proliferative outputs of tyrosine kinases, thus, restoration of PI3K/Akt and MAPK/Erk signaling is a well-established mechanism underlying resistance to targeted therapeutics [19]. In order to clarify whether MDM2-induced Osimertinib resistance follows this dogma, we treated NSCLC cells with increasing concentrations of Osimertinib and determined the phosphorylation state of Akt and Erk. As shown in Fig. 2G and Fig. S2G-S2H, Osimertinib dose-dependently suppressed the phosphorylation of EGFR, along with efficient suppression of PI3K/Akt and MAPK/Erk signaling, in NSCLC cells infected with a NC lentivirus. Immunoblotting analysis of cellular extracts from MDM2 overexpressing cells showed that Osimertinib treatment also blocked EGFR phosphorylation, as well as Akt and Erk phosphorylation, to a comparable level. These findings implied that MDM2 may drive resistance to Osimertinib without restoring PI3K/Akt and MAPK/Erk signaling, and it was convincible to believe that overexpression of MDM2 minimally augmented cell proliferation. Indeed, the cell proliferation EdU fluorescence labeling assay showed that Osimertinib suppressed the EdU-positive signal in MDM2 overexpressing cells to a similar magnitude of that in NC cells (Fig. 2H, Fig. S2I-S2J). Thus, MDM2 aberration promoted resistance to Osimertinib independent of PI3K/Akt and MAPK/Erk machinery.

### MDM2 arrests cell apoptosis through stabilization of MCL-1 protein

**Fig. 3:**
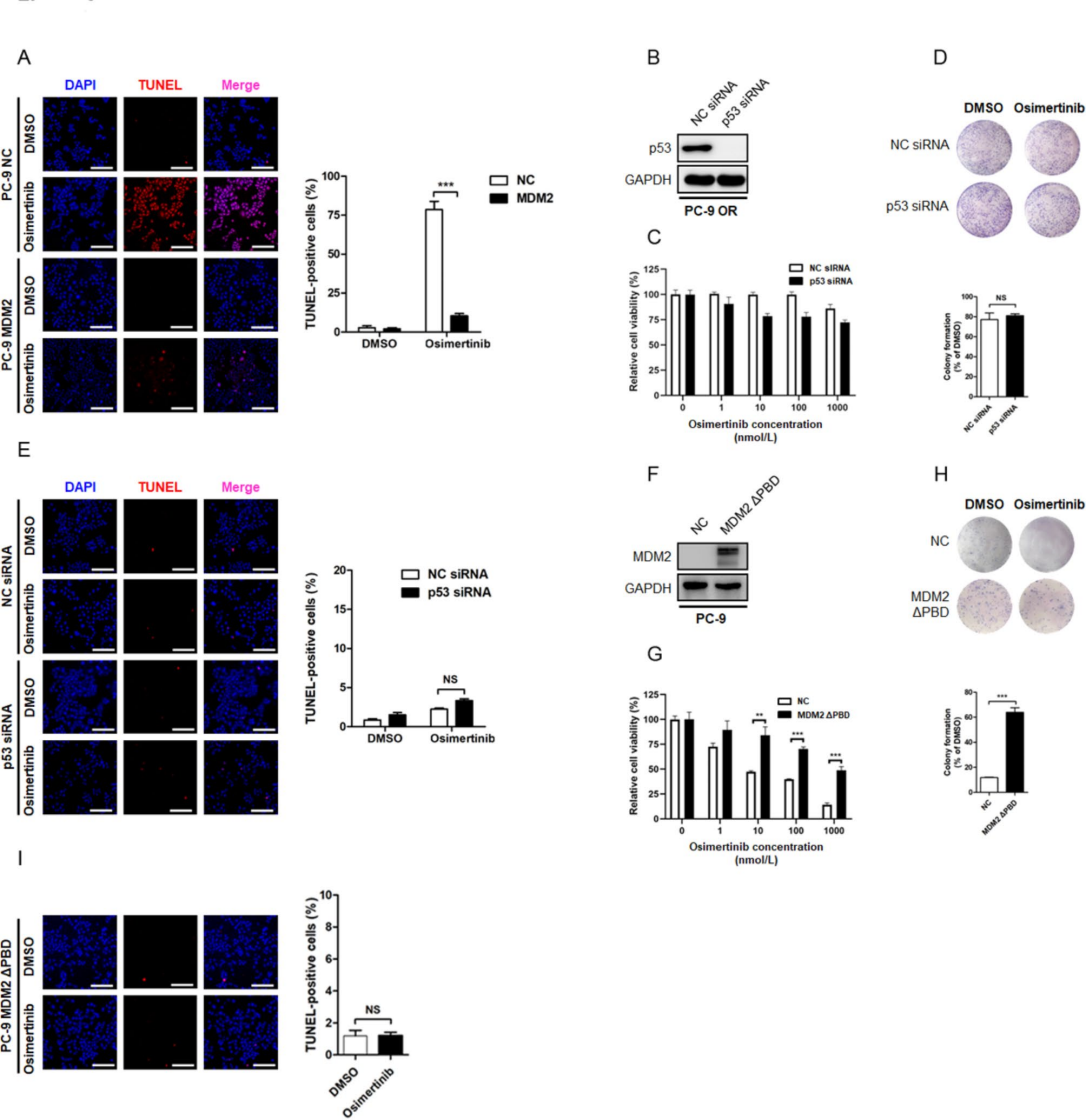
**MDM2 drives resistance to Osimertinib independent of the canonical MDM2-p53 regulatory loop. (A)** Representative fluorescent images of TUNEL assay. PC-9 NC and PC-9 MDM2 cells were treated with 1 µmol/L Osimertinib or equal amount of DMSO for 24 h. Cell apoptosis was visualized and calculated by TUNEL assay. NS: not significant. Scale bar = 200 μm. ***P < 0.001. **(B-E)** The Osimertinib resistant PC-9 OR cells were transfected with siRNA targeting p53 to deplete endogenous p53 expression and evaluated for the sensitivity to Osimertinib by MTT assay, colony formation assay, and TUNEL assay, respectively. Scale bar = 200 μm. NS: not significant. **(F-I)** The Osimertinib sensitive PC-9 cells were engineered to stably express the MDM2 ΔPBD mutant that was unable to interact with p53. The resultant cells were treated with Osimertinib or DMSO. Therapeutic response to Osimertinib was determined by cytotoxic assays, including MTT, colony formation and TUNEL assay. Scale bar = 200 μm. NS: not significant. **P < 0.01. ***P < 0.001

Aside from enhanced cell proliferation, defects in apoptosis program also enable cancer cells to escape targeted therapy-induced cell death. On the basis of the fact that MDM2 overexpression did not affect cancer cell proliferation, we sought to determine the apoptotic state in Osimertinib resistant NSCLC cells. In response to Osimertinib treatment, the sensitive cells underwent pronounced apoptosis, as measured by TUNEL assay, whereas the proportion of TUNEL-positive signal in PC-9 OR and other MDM2 overexpressing resistant cells was largely decreased (Fig. 3A, Fig. S2K-S2M). Because p53 participates in most biological events driven by MDM2, we therefore investigated whether the arrest in apoptosis was affected intracellular p53 content. We found that depletion of endogenous p53 in PC-9 OR cells minimally affected the sensitivity to Osimertinib (Fig. 3B–E). When the sensitive PC-9 cells were engineered to express a MDM2 truncated mutant that lacked the p53 binding domain (MDM2 ΔPBD), the resultant cells were still resistant to Osimertinib-induced apoptosis (Fig. 3G and I). As such, aberrant apoptotic response may contribute to Osimertinib resistance driven by MDM2, which was not likely caused by the canonical MDM2-p53 protein regulatory loop.

**Fig. 4:**
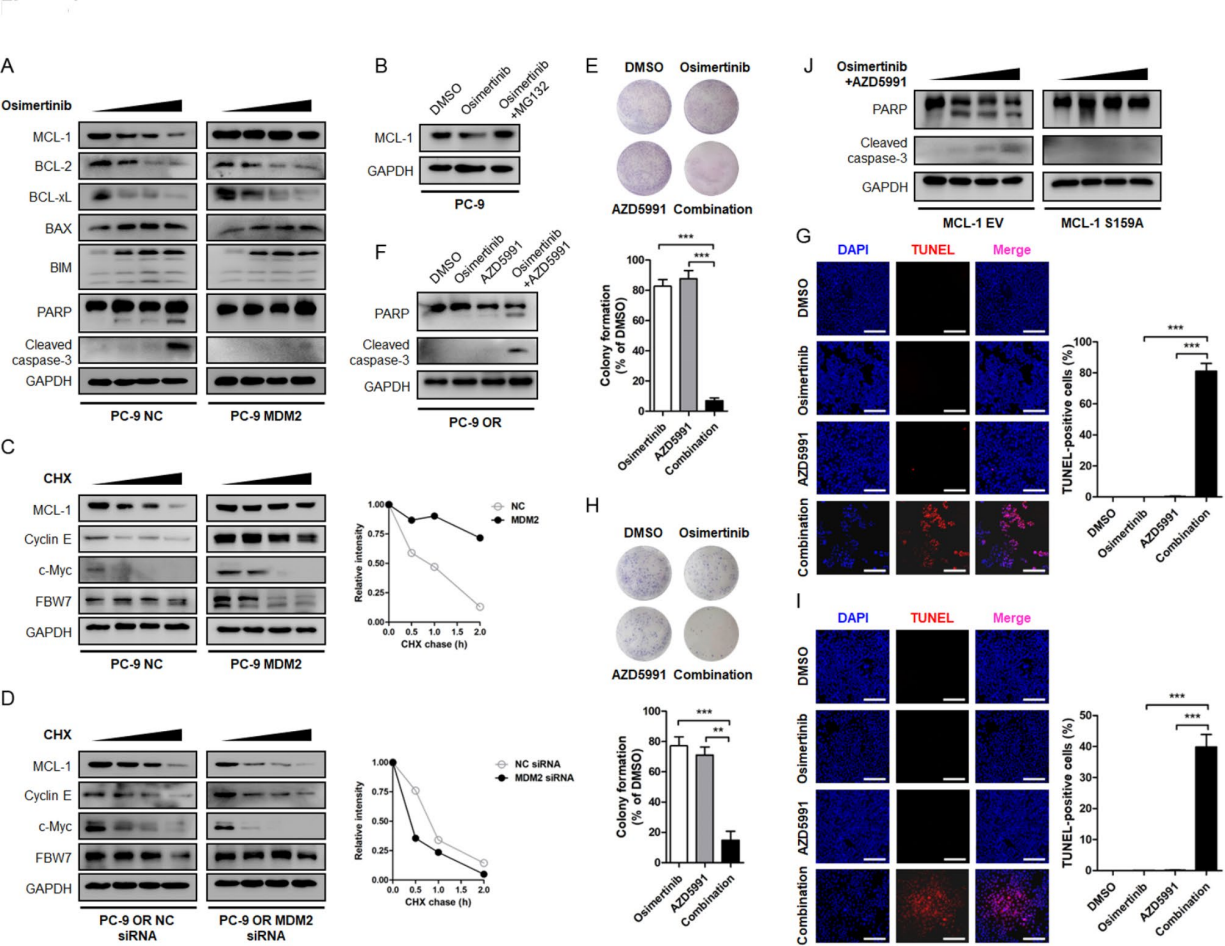
**MDM2 arrests Osimertinib-induced cancer cell apoptosis through manipulating MCL-1. (A)** PC-9 NC and PC-9 MDM2 cells were treated with increasing concentrations of Osimertinib (0, 0.1, 0.5, 1 µmol/L) for 48 h. Cell apoptosis indicators (PARP and Cleaved caspase-3) and BCL-2 family proteins involved in cell apoptosis program (MCL-1, BCL-2, BCL-xL, BAX, BIM) were analyzed by immunoblotting. GAPDH was used as an equal loading control. **(B)** PC-9 cells were treated with DMSO or 1 µmol/L Osimertinib for 48 h. To block proteasome-mediated protein degradation, MG132 at a final concentration of 20 µmol/L was added into the cell culture medium 8 h before cell harvest. MCL-1 protein level was determined by Western blot. **(C)** PC-9 MDM2 cells and PC-9 NC cells were split into 6 cm cell culture dishes and treated with 25 µg/mL CHX for indicated time intervals. Proteins of interest were separated by electrophoresis, transferred to nitrocellulose membrane and probed with indicated antibodies. **(D)** PC-9 OR cells were transfected with siRNA targeting MDM2 or NC. The half-life of candidate proteins was assessed by CHX chase assay. **(E)** Representative images of colony formation assay in PC-9 OR cells treated with DMSO, Osimertinib, AZD5991 or their combination. **(F)** Western blot analysis of PARP and caspase-3 fragmentation after 48 h of indicated treatment. **(G)** Evaluation of PC-9 OR cell apoptosis by TUNEL assay. The magnitude of apoptosis was calculated by the percentage of TUNEL-positive cells. Scale bar = 200 μm. **(H-I)** Cytotoxicity assessment of PC-9 MDM2 cells treated with DMSO, Osimertinib, AZD5991 or their combination. Scale bar = 200 μm. **(J)** Transient expression of the degradation resistant MCL-1 S159A mutant and its effect on cancer cell apoptosis induction in PC-9 OR cells treated with the Osimertinib + AZD5991 combinational strategy. **P < 0.01. ***P < 0.001

To clarify this aberrant apoptotic response, we probed cell lysates with antibodies targeting BCL-2 family members known to facilitate apoptosis. Immunoblotting assay showed that Osimertinib treatment dose-dependently triggered a decline in anti-apoptotic proteins MCL-1, BCL-2 and BCL-xL in sensitive cells, together with increased expression of pro-apoptotic protein BAX, BIM and the fragmentation of caspase-3 and PARP. In PC-9 OR and other MDM2 overexpressing resistant cells, it was noted that Osimertinib suppressed BCL-2 and BCL-xL, and increased BAX and BIM protein, to a similar magnitude compared with sensitive cells. Notably, the MCL-1 anti-apoptotic protein was refractory to Osimertinib treatment and there was no detectable reduction in MCL-1 protein abundance following treatment (Fig. 4A, Fig. S2N, Fig. S3A-S3B). Because prompt MCL-1 protein destruction was crucial for apoptosis induction [20], we speculated that MCL-1 stabilization in these MDM2 overexpressing cells mediated resistance to Osimertinib.

In support of this idea, we found Osimertinib primarily repressed MCL-1 protein through the ubiquitin-proteasome system, which could be restored by a proteasome inhibitor MG132 (Fig. 4B). The cycloheximide (CHX) chase experiments revealed that MCL-1 protein in sensitive cells was rapidly destabilized upon Osimertinib treatment, and its abundance drop to half of baseline within 2 h after stalling protein synthesis. Importantly, MDM2 antagonized Osimertinib-induced MCL-1 protein destruction and extended its half-life to different degrees (Fig. 4C, Fig. S3C-S3D), whereas depletion of endogenous MDM2 in PC-9 OR cells accelerated the degradation of MCL-1 protein (Fig. 4D). These results indicated MDM2 was required for the stabilization of MCL-1. To assess whether the stabilization of MCL-1 protein was sufficient to confer Osimertinib resistance, we treated PC-9 OR and PC-9 MDM2 cells with a combination of AZD5991, a selective and potent MCL-1 inhibitor [21]. While most Osimertinib or AZD5991 single agent-treated resistant cells remained alive and formed cell colonies, a significant percentage of cells underwent apoptosis upon their combination (Fig. 4E and I, Fig. S3E-S3F). In contrast, transient expression of a degradation resistant MCL-1 S159A mutant in PC-9 OR cells arrested cell apoptosis even in the presence of combinational treatment (Fig. 4J). These data implied that MDM2 arrested Osimertinib-induced cancer cell apoptosis at the step of defected MCL-1 destruction.

### Overcoming osimertinib resistance by dual inhibition of EGFR and MDM2

**Fig. 5:**
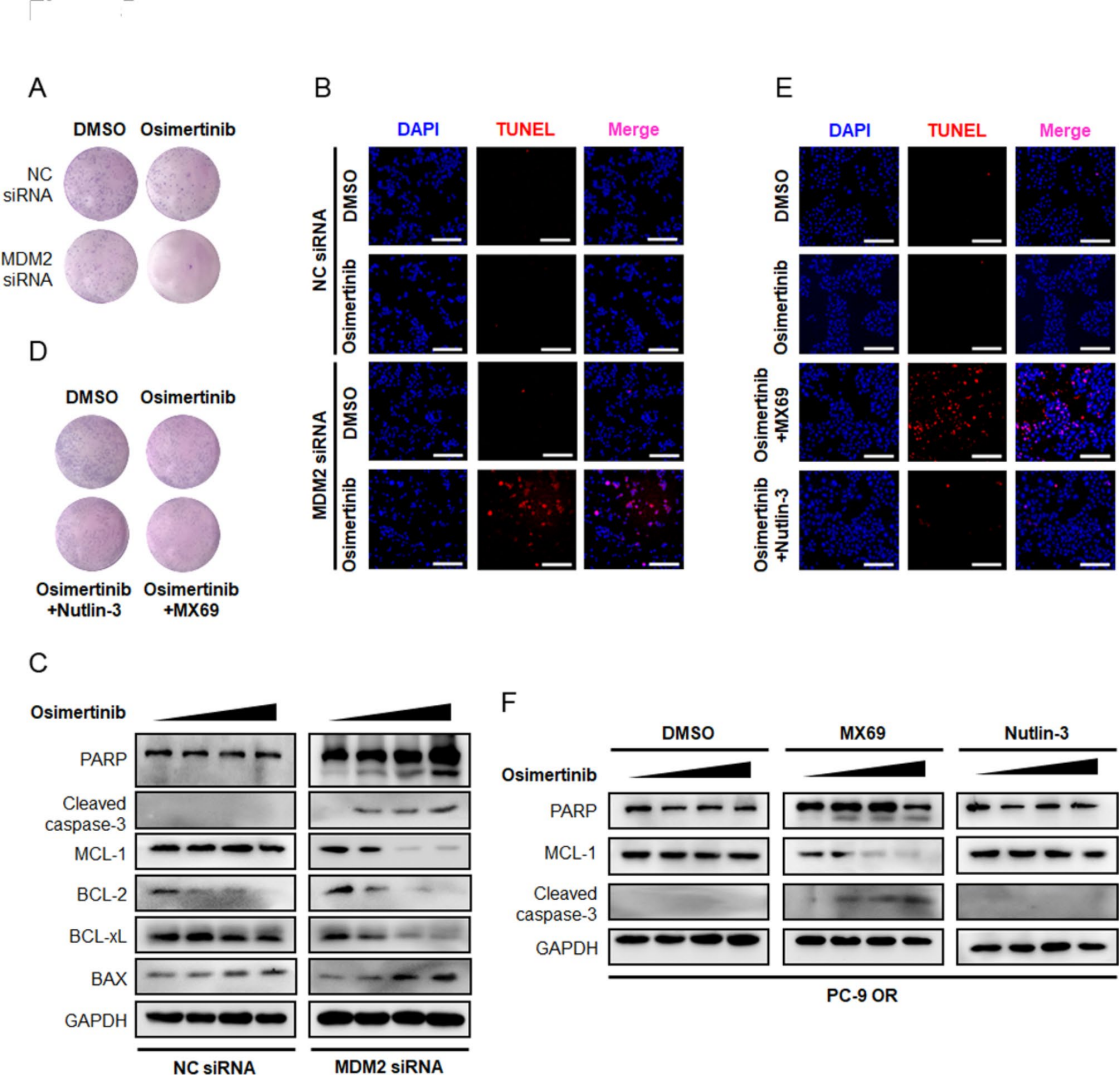
**Overcoming resistance to Osimertinib by targeting MDM2. (A-B)** The PC-9 OR cells were seeded in 6 well plate and transfected with siRNA targeting MDM2 or NC. Representative images of colony formation assay and TUNEL assay after DMSO or Osimertinib treatment were shown. Scale bar = 200 μm. **(C)** The PC-9 OR cells transfected with MDM2 siRNA or NC siRNA were treated with increasing concentrations of Osimertinib (0, 0.1, 0.5, 1 µmol/L) for 48 h. Expression of indicated BCL-2 family members and fragmentation of PARP and caspase-3 were analyzed by immunoblotting. GAPDH was used as an equal loading control. **(D-E)** The PC-9 OR cells were seeded in 6 well plate and treated with DMSO, Osimertinib, Osimertinib + Nutlin-3, and Osimertinib + MX69 for 10 days. Representative images of colony formation assay and TUNEL assay after treatment were shown. Scale bar = 200 μm. **(F)** The PC-9 OR cells were treated with Osimertinib (0, 0.1, 0.5, 1 µmol/L) and MDM2 inhibitors (5 µmol/L Nutlin-3 or 5 µmol/L MX69) for 48 h. The apoptosis state and MCL-1 protein level were assessed by immunoblotting

Although inhibiting MCL-1 could overcome resistance to Osimertinib in our experimental setting, the clinical application of MCL-1 inhibitors is challenging due to undesirable hematologic and cardiac toxicity [22]. We therefore sought to answer whether concurrently targeting EGFR and MDM2 as an alternative strategy to overcome Osimertinib resistance. We knockdown endogenous MDM2 in PC-9 OR cells with siRNAs oligos and found Osimertinib perturbed cell viability and led to a noticeably reduction in colony number (Fig. 5A). Results from TUNEL assay also yielded cancer cells responded to Osimertinib and underwent apoptosis after MDM2 depletion (Fig. 5B). Immunoblotting analysis showed the reversal of resistance was accompanied by MCL-1 destabilization (Fig. 5C), suggesting that MDM2 launched a defense mechanism against MCL-1 destruction in response to Osimertinib.

Small molecule inhibitors targeting MDM2 expression, such as MX69 (Fig. S4A), and inhibitors targeting MDM2-p53 interaction, such as Nutlin-3, have been developed. To evaluated the feasibility of pharmacologically targeting MDM2 to overcome resistance, we treated PC-9 OR and PC-9 MDM2 resistant cells with a combination of MDM2 inhibitors. Intriguingly, MDM2-conferred resistance was only reversed by MX69 (Fig. 5D and E, Fig. S4B-S4C), which dissected MDM2 and invigorated MCL-1 destruction to boost an apoptotic response to Osimertinib. While Nutlin-3 mechanistically bound to the hydrophobic pocket domain of MDM2 to selectively disrupt its interaction with p53, leaving MCL-1 protein intact and functional (Fig. 5F, Fig. S4D), failed to reverse resistance. These features heightened the necessity of rational MDM2 inhibitors selection and strategy design for effective combinations.

### The MDM2 E3 ubiquitin ligase and osimertinib resistance converge at FBW7

**Fig. 6:**
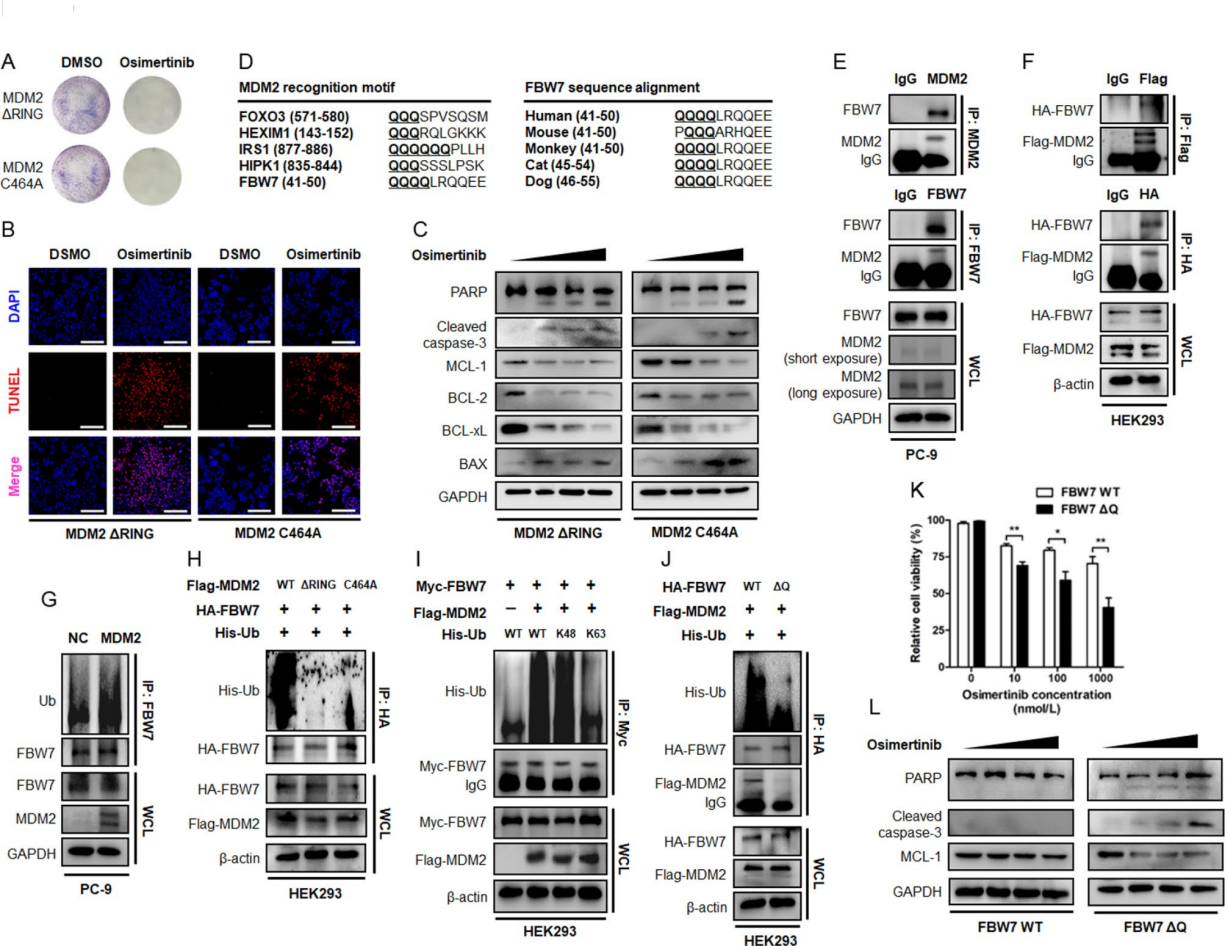
**MDM2 as a potential E3 ubiquitin ligase for FBW7 tumor suppressor. (A-B)** The parental PC-9 cells were engineered to stably express the MDM2 ΔRING or MDM2 C464A mutants that lacked E3 ubiquitin ligase activity. Cells were seeded in 6 well plate and treated with DMSO or Osimertinib. Representative images of colony formation assay and TUNEL assay were shown. Scale bar = 200 μm. **(C)** PC-9 cells stably expressing MDM2 ΔRING or MDM2 C464A mutants were treated with increasing concentrations of Osimertinib (0, 0.1, 0.5, 1 µmol/L) for 48 h. Expression of indicated BCL-2 family members and fragmentation of PARP and caspase-3 were analyzed by immunoblotting. GAPDH was used as an equal loading control. **(D)** Sequence alignment of MDM2 recognition motif across FOXO3, HEXIM1, IRS1, HIPK1 and FBW7. The conservation of Q-enriched domain in the N-terminal proportion of FBW7 was assessed across different species. **(E)** The WCL of PC-9 cells was precipitated with anti-MDM2/anti-FBW7 antibody or equal amount of isotype IgG. The resultant immunoprecipitates were subjected to immunoblotting. **(F)** HEK293 cells were transfected with HA-FBW7 together with Flag-MDM2 construct. Forty-eight hours after transfection, WCL was prepared and precipitated with anti-HA/anti-Flag antibody or equal amount of isotype IgG. The cell lysates and immunoprecipitates were subjected to immunoblotting. β-actin was used as an equal loading control. **(G)** The effect of MDM2 on the ubiquitination status of FBW7. MDM2 was stably expressed in PC-9 cells. The ubiquitination level of FBW7 was determined by immunoprecipitation with an anti-FBW7 antibody and probed with an anti-Ub antibody. **(H)** HA-FBW7 and His-Ub, together with Flag MDM2 or its ΔRING and C464A mutant were transiently expressed in HEK293 cells. HA-FBW7 protein was pull down by an anti-HA antibody and probed with an anti-His antibody. **(I)** Myc-FBW7 and Flag-MDM2, together with His-Ub or its K48-only and K63-only construct were transfected into HEK293 cells. Forty-eight hours after transfection, Myc-FBW7 protein was precipitated with an anti-Myc antibody and probed with an anti-His antibody. **(J)** Flag-MDM2, His-Ub and HA-FBW7 or its ΔQ mutant were expressed in HEK293 cells. The ubiquitination status of HA-FBW7 was determined by immunoprecipitation. **(K-L)** The PC-9 OR cells stably expressing FBW7 or its ΔQ mutant were treated with increasing concentrations of Osimertinib and evaluated for cytotoxicity by MTT assay and apoptosis immunoblotting assay, respectively. *P < 0.05. **P < 0.01

The C-terminal RING domain possesses E3 ubiquitin ligase activity and is required for the proteolytic efficacy of MDM2. In our study, overexpression of MDM2 antagonized MCL-1 protein turnover, which seemed to be in sharp contrast to its pro-proteolytic activity. To gain insight into the biological event elucidating how MDM2 engaged MCL-1 protein stabilization, we generated the MDM2 ΔRING truncated mutant and the C464A mutant within the C-terminal RING domain that abolished its E3 ubiquitin ligase activity. Ectopic expression of these constructs defective in E3 ligase activity failed to induce a resistant phenotype, as judged by a panel of cytotoxicity assays (Fig. 6A and B). Immunoblotting analysis further demonstrated that cells underwent substantial apoptosis, together with accelerated MCL-1 proteolysis, when MDM2 was replaced by its E3 ligase activity deficient mutants (Fig. 6C). Thus, the E3 ubiquitin ligase activity of MDM2 was required for maintaining an Osimertinib resistant phenotype through stabilization of MCL-1 protein.

We speculated that proteins complexed and ubiquitinated by MDM2 may be responsible for MCL-1 proteolysis. This was motivated by previous reports describing MCL-1 protein as a substrate of FBW7 E3 ubiquitin ligase, by which FBW7 directly ubiquitinates MCL-1 protein for degradation [20, 23]. It was therefore proposed that MDM2 E3 ubiquitin ligase may stabilize MCL-1 protein through manipulating FBW7 E3 ubiquitin ligase. To explore whether the interaction of MDM2 and FBW7 constituted the mechanistic basis for MDM2-mediated MCL-1 stabilization and Osimertinib resistance in NSCLC, we screened in silico prediction and literature reports and noticed a consensus recognition motif enriched in glutamine (Q) across a panel of MDM2 substrates. We grouped FBW7 amino acid sequence and found the Q-enriched motif within the N-terminal region was highly conserved among the orthologues in different vertebrate species (Fig. 6D), raising a possibility that MDM2 may restore MCL-1 protein abundance through binding and catalyzing FBW7 destruction. In agreement with this idea, we detected co-precipitation of endogenous MDM2 when FBW7 was captured by an antibody for IP. When reciprocal experiment was performed, FBW7 was pulled down and complexed with MDM2 (Fig. 6E, Fig. S5A-S5B). Likewise, Flag-tagged MDM2 and HA-tagged FBW7 were found to complex with each other upon transient expression in HEK293 cells (Fig. 6F). The binding to MDM2 resulted in K48-linked polyubiquitination, but not K63-linked polyubiquitination, of FBW7 protein (Fig. 6G and I, Fig. S5C-S5D), whereas FBW7 engineered to lose the Q-enriched consensus motif (ΔQ) failed to complex with MDM2 as efficiently as FBW7 (Fig. 6J). To this end, it is conceivable that ectopic expression of the ubiquitination resistant FBW7 ΔQ mutant in PC-9 OR resistant cells ensured prompt MCL-1 proteolysis and rescued sensitivity to Osimertinib (Fig. 6K and L, Fig. S5E).

### Biological interaction between MDM2 and FBW7 and its clinical relevance

**Fig. 7:**
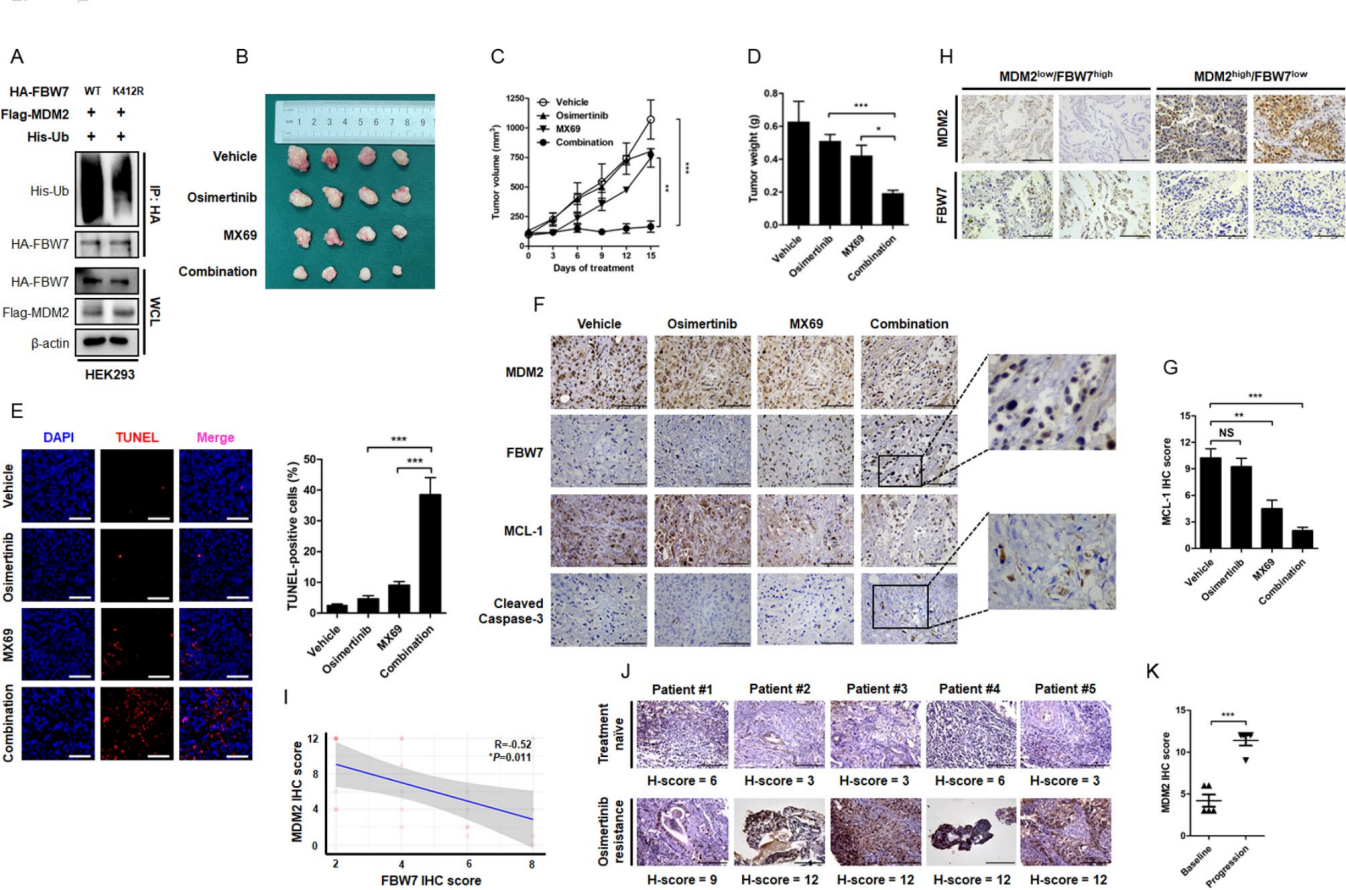
**Targeting MDM2 is a feasible approach to overcome resistance to Osimertinib in vivo and its clinical relevance during Osimertinib progression. (A)** HA-FBW7 or its K412R mutant was expressed in HEK293 cells. The ubiquitination status of FBW7 was determined by immunoprecipitation assay. **(B-D)** Representative images of PC-9 OR cells xenograft tumors grew in nude mice receiving Vehicle, Osimertinib (5 mg/kg), MX69 (20 mg/kg) and their combination. The tumor volume was routinely monitored. At the end of the experiment, the xenograft tumors were carefully removed, weighted and photographed. *P < 0.05, **P < 0.01, ***P < 0.001. **(E)** Evaluation of cell apoptosis in xenograft tumors by TUNEL assay. Scale bar = 200 μm. ***P < 0.001. **(F)** IHC staining of MDM2, FBW7, MCL-1 and cleaved caspase-3 in xenograft tumors. The enlarged window indicated protein intensity and distribution pattern. Scale bar = 100 μm. **(G)** Analysis of MCL-1 IHC score after indicated treatment. NS: not significant. **P < 0.01, ***P < 0.001. **(H-I)** A total number of 23 cases of surgical resected NSCLC were analyzed for MDM2 and FBW7 expression by IHC staining. Scale bar = 100 μm. Each NSCLC sample yielded an IHC score for MDM2 and FBW7, respectively. The correlation between MDM2 and FBW7 was determined by linear regression analysis. **(J-K)** IHC analysis of MDM2 expression in matched treatment-naïve and Osimertinib resistant NSCLC patients. Scale bar = 100 μm. NS: not significant. ***P < 0.001

Consistent with a role as E3 ubiquitin ligase, overexpression of MDM2 accelerated the turnover of FBW7 protein in multiple NSCLC cell lines, along with the stabilization of proteins targeted by FBW7, such as MCL-1, cyclin E and c-Myc (Fig. 4C). In contrast, depletion of endogenous MDM2 enhanced FBW7 stability and prompted degradation of MCL-1, cyclin E and c-Myc (Fig. 4D), supporting the physiological role of MDM2 in regulating FBW7 protein abundance and biological functions. To precisely map the MDM2 ubiquitination site on FBW7, we used computational prediction that yielded the evolutionarily conserved Lys412 residue within C-terminal WD40 domain as a putative ubiquitination site targeted by MDM2. To validate this site of interest, we generated the FBW7 K412R mutant for ubiquitination analysis. Notably, the ubiquitination state of FBW7 was largely compromised when the Lysine was replaced by Arginine (Fig. 7A), supporting the Lys412 residue is indeed a major site for MDM2-mediated ubiquitination of FBW7.

**Fig. 8:**
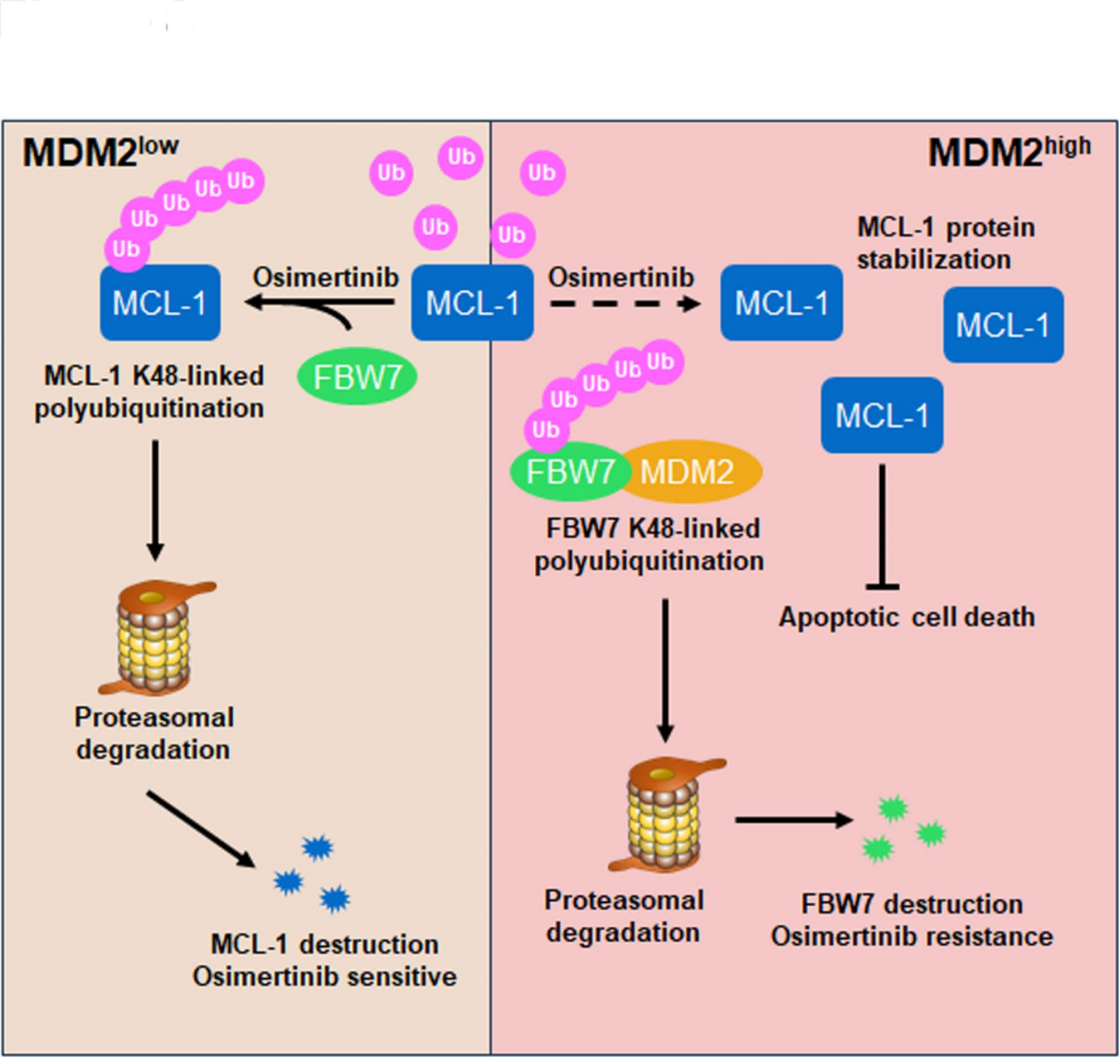
**A schematic diagram of MDM2-mediated resistance machinery and its implications for overcoming resistance to Osimertinib in EGFR mutant NSCLC.** In EGFR mutant/MDM2^low^ NSCLC, Osimertinib treatment triggers MCL-1 anti-apoptotic protein ubiquitination catalyzed by FBW7 E3 ligase that leads to MCL-1 protein destruction and substantial apoptosis. These MDM2^low^ tumors manifest as Osimertinib sensitive NSCLC. However, when cancer cells gain high expression of MDM2, MDM2 acts as an E3 ubiquitin ligase for FBW7 to degrade this tumor suppressor, which results in the accumulation of MCL-1 anti-apoptotic protein. MCL-1 in MDM2^high^ tumors is refractory to FBW7-mediated protein destruction and therefore cancer cells are resistant to Osimertinib-induced apoptotic cell death. Hence, targeting MDM2 to augment prompt destruction of MCL-1 protein would be a promising strategy to overcome resistance to Osimertinib in EGFR mutant NSCLC

Finally, we investigated the therapeutic efficacy of co-targeting MDM2 to overcome resistance to Osimertinib in vivo. PC-9 OR cells were subcutaneously seeded in nude mice and treated with Osimertinib, MX69, or their combination. It was noted that Osimertinib elicited very limited single agent anti-tumor efficacy to Osimertinib since the xenograft tumor continued to grow in the presence of Osimertinib and the tumor volume and tumor weight in the Osimertinib group was comparable to that in Vehicle group. Treatment with MX69 revealed modest anti-tumor activity with a slight decrease in tumor size and a slight increase in tumor weight. However, a significant reduction in xenograft tumor outgrowth was noticed when these two drugs were combined with each other (Fig. 7B–D). This combinational strategy was the most effective in reducing tumor volume and weight, probably mainly through the induction of extensive apoptotic cell death (Fig. 7E). Histological evaluation of the isolated xenograft tumor showed the drug combination strategy provoked the strongest inhibitory potency on MCL-1 protein expression and the strongest capacity on apoptosis induction, together with the breakdown of MDM2 oncoprotein and the restoration of FBW7 tumor suppressor (Fig. 7F and G). Indeed, the reverse correlation between MDM2 and FBW7 was clinically relevant. We evaluated the expression of MDM2 and FBW7 in 23 cases of surgical resected NSCLC by IHC staining and found that the MDM2^low^ tumors tended to have a relative higher expression level of FBW7, whereas FBW7 expression in the MDM2^high^ tumors was largely diminished (Fig. 7H). The regression analysis of corresponding IHC scores confirmed a negative correlation between MDM2 and FBW7 with a *R* value of -0.52 (Fig. 7I). Hence, MDM2 is a physical negative regulator of FBW7 in NSCLC. Finally, MDM2 overexpression as a mechanism of resistance to Osimertinib has been validated in repeated biopsy samples upon disease progression. Evaluation of MDM2 expression in paired treatment-naïve and treatment-failure specimens yielded higher MDM2 IHC scores after Osimertinib resistance (Fig. 7J and K). Collectively, these findings demonstrated that FBW7 expression in NSCLC is tightly regulated by MDM2 E3 ligase. The protein interaction between MDM2 and FBW7 definitely exists in clinical setting and their balance determines the sensitivity to Osimertinib in EGFR mutant NSCLC. Disruption of the balance, such as MDM2 amplification, promotes FBW7 protein destruction and impairs the cell apoptosis program to lead resistance to targeted therapy (Fig. 8). Thus, manipulating MDM2-FBW7 interaction would be a feasible approach to overcome resistance to Osimertinib in EGFR mutant NSCLC.

## Discussion

Although secondary mutations and compensatory bypass pathways have been demonstrated to lead resistance to Osimertinib in EGFR mutant NSCLC, there is still a big gap of knowledge regarding resistance machineries. Herein, we exploited the inhibitory potency of MDM2 on the sensitivity to Osimertinib. We found patients with MDM2 amplification failed on Osimertinib treatment. Overexpression of MDM2 selectively resulted in resistance to EGFR targeted therapy, but not chemotherapy, in NSCLC (Fig. S6). Resistance driven by MDM2 is not associated with reactivation of PI3K/Akt and MAPK/Erk signaling. Instead, MDM2 disrupts cell apoptosis program through K48-linked polyubiquitination of FBW7 tumor suppressor. Combined targeting EGFR and MDM2 stabilize FBW7, as a result, accelerates prompt MCL-1 protein turnover and augments cancer cell apoptosis (Fig. 8). Thus, screening MDM2 state to select patients to receive MDM2 inhibitors would be a promising strategy to overcome resistance to Osimertinib in patients with EGFR mutant NSCLC.

Unlike the currently identified resistance mechanisms, MDM2 does not rescue the phosphorylation state of Akt and Erk, which is dominantly implicated in cell proliferation. We showed that MDM2 selectively impairs cell apoptosis process to evade targeted therapy-induced cell death. The evasion of EGFR inhibitor-induced apoptosis as a mechanism of resistance has also been validated in patient-derived MGH134 resistant cells by scientists from MGH Cancer Center. In their study, the third generation EGFR inhibitor WZ4002 readily diminished EGFR and downstream PI3K/Akt and MAPK/Erk signaling in MGH134 cells [24]. By unbiased drug screening, the authors found the resistant cells were sensitive to combined treatment with ABT-263, a dual BCL-xL and BCL-2 inhibitor, presumably by induction of pro-apoptotic BH3-only protein BIM. Interestingly, the pro-apoptotic activity of BIM is antagonized by its binding partner MCL-1, which has been extensively studied in the present study. The binding to MCL-1 sequesters BIM and blocks BIM-mediated mitochondrial apoptotic cascade [25, 26]. Prompt destruction of MCL-1 thus releases MCL-1-free BIM to initiate apoptosis signaling. In NSCLC cells with EGFR activating mutations, blockade of EGFR signaling accelerates MCL-1 degradation and triggers cancer cell apoptosis [27]. To this end, inhibition of cell survival pathways and initiation of apoptosis pathway underlie the robust clinical efficacy of EGFR inhibitors in sensitive cells. When these pathways are not coincidentally suppressed, cancers are unresponsive to targeted therapy and resistance eventually develops. Our study complements these findings, showing that Osimertinib resistance driven by MDM2 is associated with the stabilization of MCL-1 protein and the arrest in apoptosis induction. Given the importance of MCL-1 proteolysis for apoptosis priming, prompt destruction of MCL-1 as an immediate response to anti-cancer treatment therefore underlies sensitivity to Osimertinib in EGFR mutant NSCLC. MDM2-mediated stabilization of MCL-1 provides a survival advantage over Osimertinib, and thus leads to resistance to targeted therapy. Unfortunately, the MCL-1 inhibitor AZD5991 elicited limited single agent efficacy in inducing resistant cell death, probably because it does not efficiently repress cell proliferation. Hence, manipulation of protein regulators upstream of MCL-1 would be a promising strategy to overcome resistance to Osimertinib driven by MDM2 in NSCLC.

Our results provide proof-of-principle that MDM2 utilizes its E3 ubiquitin ligase activity to disrupt cancer cell apoptosis through stabilization of MCL-1 protein, independent of the canonical MDM2-p53 interaction loop. While these findings might provide a rational explanation for apoptosis evasion and drug resistance, it is confusing how the proteolytic activity affects MDM2 to stabilize MCL-1 protein. We proposed a model that protein regulators upstream of MCL-1 could be harnessed by MDM2 E3 ubiquitin ligase, and the key motivation in our study is to identify the molecule connecting MDM2 and MCL-1. We demonstrated that FBW7 E3 ubiquitin ligase might be positioned downstream of MDM2 in a signaling circuit to cooperate with MDM2 in the regulation of MCL-1 protein stability and apoptotic response to Osimertinib. Specifically, MDM2 degrades FBW7, a process that requires the E3 ubiquitin ligase activity to catalyze the labeling with ubiquitin chain to FBW7, which in return resulted in the accumulation of FBW7 substrates, such as MCL-1, to induce resistance to Osimertinib. FBW7 substrates are recruited to MDM2 by docking to FBW7 and therefore their interactions with MDM2 are indirect and less sufficient [28]. Thus, FBW7 acts as a molecular hub integrating MDM2 and MCL-1. Overexpression of MDM2 catalyzes ubiquitination and degradation of FBW7, as a consequence, stabilizes MCL-1 protein for implementing resistance to Osimertinib-induced apoptotic cell death.

In addition to providing the mechanisms underlying MDM2’s E3 ligase activity-dependent control of cell apoptosis program, our study also highlighted a novel regulatory machinery that governs intracellular FBW7 E3 ubiquitin ligase protein abundance. In sharp contrast to prolyl isomerase Pin that has been shown to promote degradation of FBW7 through increasing self-ubiquitination [29], MDM2 accelerates FBW7 protein turnover by directly augmenting K48 polyubiquitination at Lys412 residue. To this end, MDM2 is recognized as an E3 ubiquitin ligase for FBW7 E3 ubiquitin ligase, the balance between FBW7’s ubiquitination promoting enzymes (Pin, MDM2) and ubiquitination removing enzymes (USP28, USP9X) therefore maintains FBW7 homeostasis and restrains the oncogenic function of FBW7 substrates [30, 31]. Overexpression of MDM2 disrupts the balance and results in a FBW7^low^/MCL-1^high^ state, leading to defects in cell apoptosis program and resistance to Osimertinib. Targeting MDM2 by small molecule inhibitors to remodel the aberrant FBW7/MCL-1 state is expected to be a promising strategy for overcoming resistance to Osimertinib. Intriguingly, Nutlin-3 and MX69 as paradigms of two types of MDM2 inhibitors revealed different therapeutic outcome, again, highlighting the E3 ubiquitin ligase activity of MDM2 is required for driving resistance to Osimertinib. While selectively targeting MDM2’s E3 ligase activity is still technical challenging, targeted degradation of MDM2 using the emerging PROteolysis TArgeting Chimera (PROTAC) technique therefore enables a feasible approach for governing MDM2 expression and function. Several designated MDM2 PROTACs have already entered early phase clinical trials and preliminary data showed on-target anti-tumor efficacy in triple negative breast cancer [32]. It is speculated that MDM2 PROTACs may permit more efficacious engagement and selective destruction of MDM2 protein in NSCLC cells, and combining MDM2 PROTACs and targeted therapy would be a promising strategy to overcome resistance to Osimertinib in patients with EGFR mutant NSCLC.

## Conclusion

In summary, our study deciphered a novel and clinically relevant resistance mechanism to Osimertinib in EGFR mutant NSCLC. MDM2 amplification prominently destructs FBW7 tumor suppressor and stabilize MCL-1 anti-apoptotic protein to evade apoptotic cell death. Targeting MDM2 to restore the FBW7-MCL-1-apoptosis cascade would be a feasible approach to overcome resistance to targeted therapy in NSCLC.

## Electronic supplementary material

Below is the link to the electronic supplementary material.

Supplementary Material 1

Supplementary Material 2

Supplementary Material 3

Supplementary Material 4

Supplementary Material 5

Supplementary Material 6

Supplementary Material 7

Supplementary Material 8

## Data Availability

The datasets used and analyzed in this study are available from the corresponding author on reasonable request.

## Abbreviations

CHX: cycloheximide
EdU: 5-Ethynyl-2’-deoxyuridine
EGFR: epidermal growth factor receptor
FBW7: F-box/WD repeat-containing protein 7
IHC: immunohistochemistry
MCL-1: myeloid cell leukemia-1
MDM2: murine double minute 2
NC: negative control
NGS: next-generation sequencing
NSCLC: non-small cell lung cancer
PFS: progression-free survival
siRNAs: small interfere RNAs
TKIs: tyrosine kinase inhibitors
TUNEL: TdT-mediated dUTP Nick-End Labeling
WCL: whole cell lysate

## References

[1] Sordella R, Bell DW, Haber DA, Settleman J. Gefitinib-sensitizing EGFR mutations in lung cancer activate anti-apoptotic pathways. Science. 2004;305(5687):1163–7. 15284455 10.1126/science.1101637

[2] Lynch TJ, Bell DW, Sordella R, Gurubhagavatula S, Okimoto RA, Brannigan BW, Harris PL, Haserlat SM, Supko JG, Haluska FG, et al. Activating mutations in the epidermal growth factor receptor underlying responsiveness of non-small-cell lung cancer to gefitinib. N Engl J Med. 2004;350(21):2129–39. 15118073 10.1056/NEJMoa040938

[3] Mok TS, Wu YL, Thongprasert S, Yang CH, Chu DT, Saijo N, Sunpaweravong P, Han B, Margono B, Ichinose Y, et al. Gefitinib or carboplatin-paclitaxel in pulmonary adenocarcinoma. N Engl J Med. 2009;361(10):947–57. 19692680 10.1056/NEJMoa0810699

[4] Park K, Tan EH, O’Byrne K, Zhang L, Boyer M, Mok T, Hirsh V, Yang JC, Lee KH, Lu S, et al. Afatinib versus Gefitinib as first-line treatment of patients with EGFR mutation-positive non-small-cell lung cancer (LUX-Lung 7): a phase 2B, open-label, randomised controlled trial. Lancet Oncol. 2016;17(5):577–89. 27083334 10.1016/S1470-2045(16)30033-X

[5] Wu YL, Cheng Y, Zhou X, Lee KH, Nakagawa K, Niho S, Tsuji F, Linke R, Rosell R, Corral J, et al. Dacomitinib versus Gefitinib as first-line treatment for patients with EGFR-mutation-positive non-small-cell lung cancer (ARCHER 1050): a randomised, open-label, phase 3 trial. Lancet Oncol. 2017;18(11):1454–66. 28958502 10.1016/S1470-2045(17)30608-3

[6] Soria JC, Ohe Y, Vansteenkiste J, Reungwetwattana T, Chewaskulyong B, Lee KH, Dechaphunkul A, Imamura F, Nogami N, Kurata T, et al. Osimertinib in untreated EGFR-Mutated Advanced Non-small-cell Lung Cancer. N Engl J Med. 2018;378(2):113–25. 29151359 10.1056/NEJMoa1713137

[7] Mok TS, Wu YL, Ahn MJ, Garassino MC, Kim HR, Ramalingam SS, Shepherd FA, He Y, Akamatsu H, Theelen WS, et al. Osimertinib or Platinum-Pemetrexed in EGFR T790M-Positive Lung Cancer. N Engl J Med. 2017;376(7):629–40. 27959700 10.1056/NEJMoa1612674 PMC6762027

[8] Wu YL, Tsuboi M, He J, John T, Grohe C, Majem M, Goldman JW, Laktionov K, Kim SW, Kato T, et al. Osimertinib in Resected EGFR-Mutated non-small-cell Lung Cancer. N Engl J Med. 2020;383(18):1711–23. 32955177 10.1056/NEJMoa2027071

[9] Ahn MJ, Chiu CH, Cheng Y, Han JY, Goldberg SB, Greystoke A, Crawford J, Zhao Y, Huang X, Johnson M, et al. Osimertinib for patients with Leptomeningeal metastases Associated with EGFR T790M-Positive Advanced NSCLC: the AURA Leptomeningeal metastases Analysis. J Thorac Oncology: Official Publication Int Association Study Lung Cancer. 2020;15(4):637–48. 10.1016/j.jtho.2019.12.113 31887431

[10] Thress KS, Paweletz CP, Felip E, Cho BC, Stetson D, Dougherty B, Lai Z, Markovets A, Vivancos A, Kuang Y, et al. Acquired EGFR C797S mutation mediates resistance to AZD9291 in non-small cell lung cancer harboring EGFR T790M. Nat Med. 2015;21(6):560–2. 25939061 10.1038/nm.3854 PMC4771182

[11] Leonetti A, Sharma S, Minari R, Perego P, Giovannetti E, Tiseo M. Resistance mechanisms to osimertinib in EGFR-mutated non-small cell lung cancer. Br J Cancer. 2019;121(9):725–37. 31564718 10.1038/s41416-019-0573-8 PMC6889286

[12] Sequist LV, Han JY, Ahn MJ, Cho BC, Yu H, Kim SW, Yang JC, Lee JS, Su WC, Kowalski D, et al. Osimertinib plus Savolitinib in patients with EGFR mutation-positive, MET-amplified, non-small-cell lung cancer after progression on EGFR tyrosine kinase inhibitors: interim results from a multicentre, open-label, phase 1b study. Lancet Oncol. 2020;21(3):373–86. 32027846 10.1016/S1470-2045(19)30785-5

[13] Marcoux N, Gettinger SN, O’Kane G, Arbour KC, Neal JW, Husain H, Evans TL, Brahmer JR, Muzikansky A, Bonomi PD, et al. EGFR-Mutant Adenocarcinomas that transform to small-cell Lung Cancer and other neuroendocrine carcinomas: clinical outcomes. J Clin Oncology: Official J Am Soc Clin Oncol. 2019;37(4):278–85. 10.1200/JCO.18.01585 PMC7001776 30550363

[14] Labbe C, Cabanero M, Korpanty GJ, Tomasini P, Doherty MK, Mascaux C, Jao K, Pitcher B, Wang R, Pintilie M, et al. Prognostic and predictive effects of TP53 co-mutation in patients with EGFR-mutated non-small cell lung cancer (NSCLC). Lung Cancer. 2017;111:23–9. 28838393 10.1016/j.lungcan.2017.06.014

[15] Elkrief A, Odintsov I, Markov V, Caeser R, Sobczuk P, Tischfield SE, Bhanot U, Vanderbilt CM, Cheng EH, Drilon A et al. Combination therapy with MDM2 and MEK inhibitors is effective in patient-derived models of lung adenocarcinoma with concurrent oncogenic drivers and MDM2 amplification. J Thorac Oncology: Official Publication Int Association Study Lung Cancer 2023. 10.1016/j.jtho.2023.05.007 PMC10524759 37182602

[16] Wade M, Li YC, Wahl GM. MDM2, MDMX and p53 in oncogenesis and cancer therapy. Nat Rev Cancer. 2013;13(2):83–96. 23303139 10.1038/nrc3430 PMC4161369

[17] Marine JC, Lozano G. Mdm2-mediated ubiquitylation: p53 and beyond. Cell Death Differ. 2010;17(1):93–102. 19498444 10.1038/cdd.2009.68

[18] Ye M, Zhang Y, Zhang X, Zhang J, Jing P, Cao L, Li N, Li X, Yao L, Zhang J, et al. Targeting FBW7 as a strategy to overcome resistance to targeted therapy in Non-small Cell Lung Cancer. Cancer Res. 2017;77(13):3527–39. 28522751 10.1158/0008-5472.CAN-16-3470

[19] Engelman JA. Targeting PI3K signalling in cancer: opportunities, challenges and limitations. Nat Rev Cancer. 2009;9(8):550–62. 19629070 10.1038/nrc2664

[20] Inuzuka H, Shaik S, Onoyama I, Gao D, Tseng A, Maser RS, Zhai B, Wan L, Gutierrez A, Lau AW, et al. SCF(FBW7) regulates cellular apoptosis by targeting MCL1 for ubiquitylation and destruction. Nature. 2011;471(7336):104–9. 21368833 10.1038/nature09732 PMC3076007

[21] Tron AE, Belmonte MA, Adam A, Aquila BM, Boise LH, Chiarparin E, Cidado J, Embrey KJ, Gangl E, Gibbons FD, et al. Discovery of Mcl-1-specific inhibitor AZD5991 and preclinical activity in multiple myeloma and acute myeloid leukemia. Nat Commun. 2018;9(1):5341. 30559424 10.1038/s41467-018-07551-w PMC6297231

[22] Eastman RSS. BCL2 inhibitors as Anticancer drugs: a plethora of misleading BH3 mimetics. Mol Cancer Ther. 2016;15(9):2011–7. 27535975 10.1158/1535-7163.MCT-16-0031 PMC5010924

[23] Wertz IE, Kusam S, Lam C, Okamoto T, Sandoval W, Anderson DJ, Helgason E, Ernst JA, Eby M, Liu J, et al. Sensitivity to antitubulin chemotherapeutics is regulated by MCL1 and FBW7. Nature. 2011;471(7336):110–4. 21368834 10.1038/nature09779

[24] Hata AN, Niederst MJ, Archibald HL, Gomez-Caraballo M, Siddiqui FM, Mulvey HE, Maruvka YE, Ji F, Bhang HE, Krishnamurthy Radhakrishna V, et al. Tumor cells can follow distinct evolutionary paths to become resistant to epidermal growth factor receptor inhibition. Nat Med. 2016;22(3):262–9. 26828195 10.1038/nm.4040 PMC4900892

[25] Han J, Goldstein LA, Gastman BR, Rabinowich H. Interrelated roles for Mcl-1 and BIM in regulation of TRAIL-mediated mitochondrial apoptosis. J Biol Chem. 2006;281(15):10153–63. 16478725 10.1074/jbc.M510349200

[26] Niu X, Zhao J, Ma J, Xie C, Edwards H, Wang G, Caldwell JT, Xiang S, Zhang X, Chu R, et al. Binding of released bim to Mcl-1 is a mechanism of intrinsic resistance to ABT-199 which can be overcome by combination with Daunorubicin or cytarabine in AML cells. Clin cancer Research: Official J Am Association Cancer Res. 2016;22(17):4440–51. 10.1158/1078-0432.CCR-15-3057 PMC5010519 27103402

[27] Shi P, Oh YT, Deng L, Zhang G, Qian G, Zhang S, Ren H, Wu G, Legendre B Jr., Anderson E, et al. Overcoming Acquired Resistance to AZD9291, a third-generation EGFR inhibitor, through Modulation of MEK/ERK-Dependent Bim and Mcl-1 degradation. Clin cancer Research: Official J Am Association Cancer Res. 2017;23(21):6567–79. 10.1158/1078-0432.CCR-17-1574 PMC5668147 28765329

[28] Wunderlich M, Berberich SJ. Mdm2 inhibition of p53 induces E2F1 transactivation via p21. Oncogene. 2002;21(28):4414–21. 12080472 10.1038/sj.onc.1205541

[29] Min SH, Lau AW, Lee TH, Inuzuka H, Wei S, Huang P, Shaik S, Lee DY, Finn G, Balastik M, et al. Negative regulation of the stability and tumor suppressor function of Fbw7 by the Pin1 prolyl isomerase. Mol Cell. 2012;46(6):771–83. 22608923 10.1016/j.molcel.2012.04.012 PMC3389221

[30] Khan OM, Carvalho J, Spencer-Dene B, Mitter R, Frith D, Snijders AP, Wood SA, Behrens A. The deubiquitinase USP9X regulates FBW7 stability and suppresses colorectal cancer. J Clin Investig. 2018;128(4):1326–37. 29346117 10.1172/JCI97325 PMC5873885

[31] Schulein-Volk C, Wolf E, Zhu J, Xu W, Taranets L, Hellmann A, Janicke LA, Diefenbacher ME, Behrens A, Eilers M, et al. Dual regulation of Fbw7 function and oncogenic transformation by Usp28. Cell Rep. 2014;9(3):1099–109. 25437563 10.1016/j.celrep.2014.09.057

[32] Adams CM, Mitra R, Xiao Y, Michener P, Palazzo J, Chao A, Gour J, Cassel J, Salvino JM, Eischen CM. Targeted MDM2 degradation reveals a new vulnerability for p53-Inactivated triple-negative breast Cancer. Cancer Discov. 2023;13(5):1210–29. 36734633 10.1158/2159-8290.CD-22-1131 PMC10164114

