# Supplementary figures and images for: MDM2 drives resistance to Osimertinib by contextually disrupting FBW7-mediated destruction of MCL-1 protein in EGFR mutant NSCLC
Source: J Exp Clin Cancer Res. 2024 Nov 15;43:302. doi: 10.1186/s13046-024-03220-7 (PMC11566350; doi:10.1186/s13046-024-03220-7)

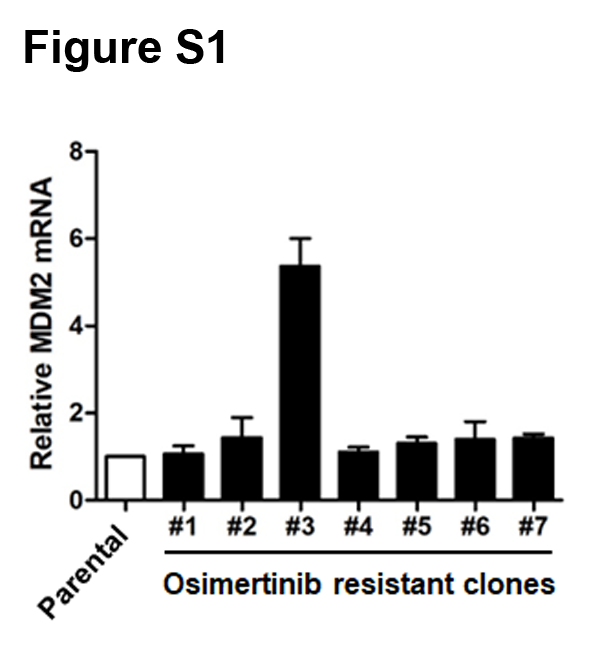

Supplement: Supplementary file 1 — Supplementary Material 1 [file 13046_2024_3220_MOESM1_ESM.tif]

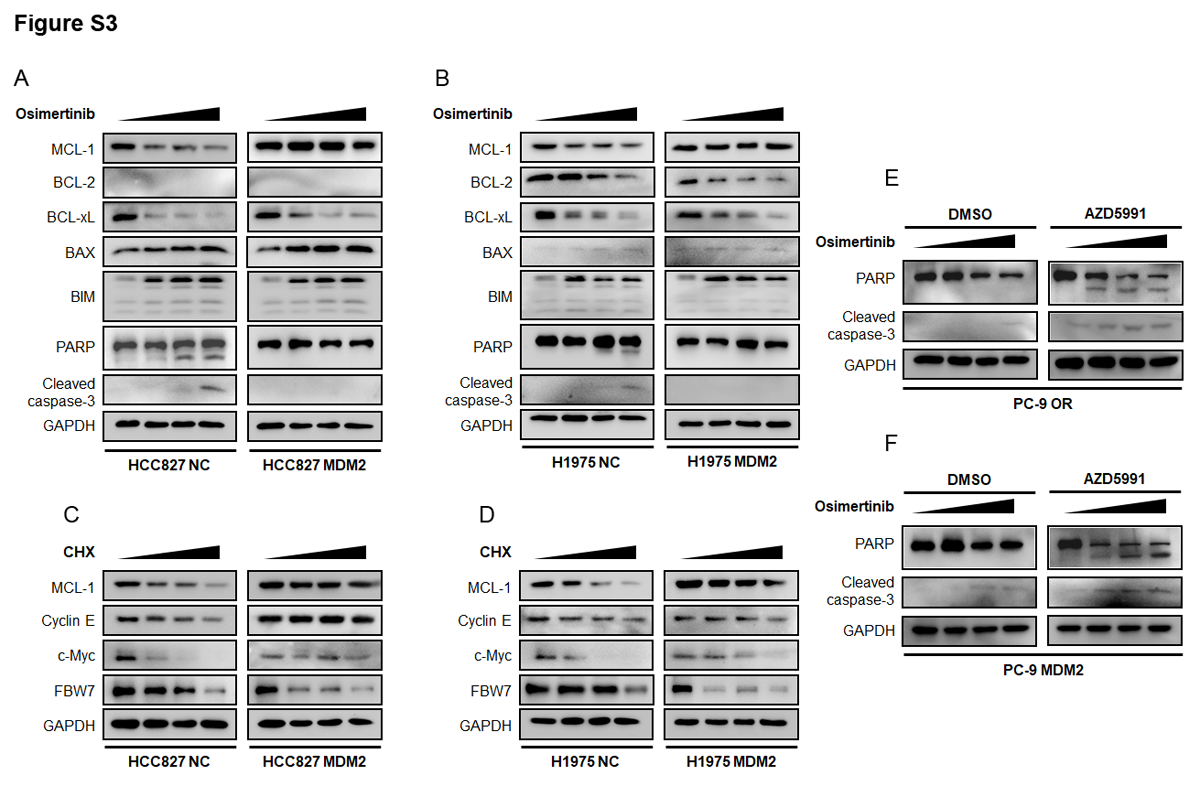

Supplement: Supplementary file 2 — Supplementary Material 2 [file 13046_2024_3220_MOESM2_ESM.tif]

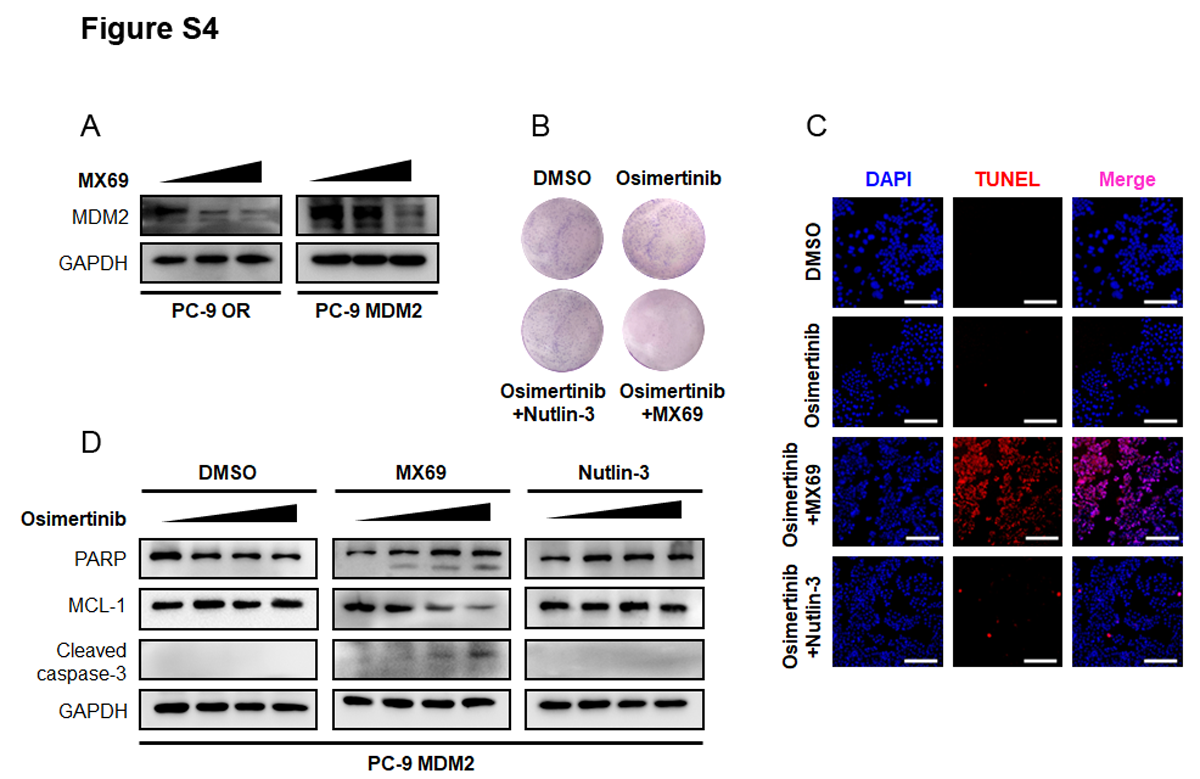

Supplement: Supplementary file 3 — Supplementary Material 3 [file 13046_2024_3220_MOESM3_ESM.tif]

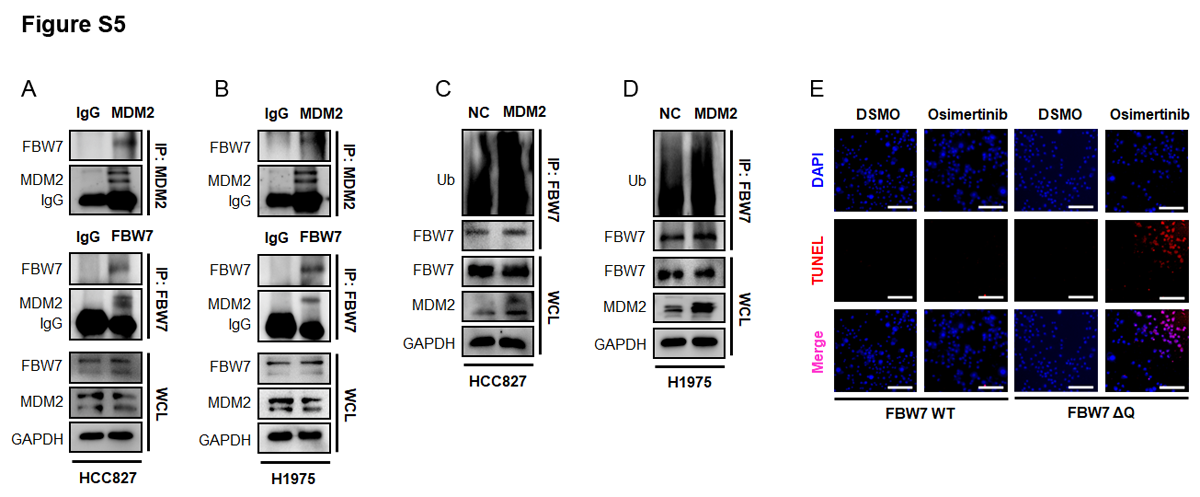

Supplement: Supplementary file 4 — Supplementary Material 4 [file 13046_2024_3220_MOESM4_ESM.tif]

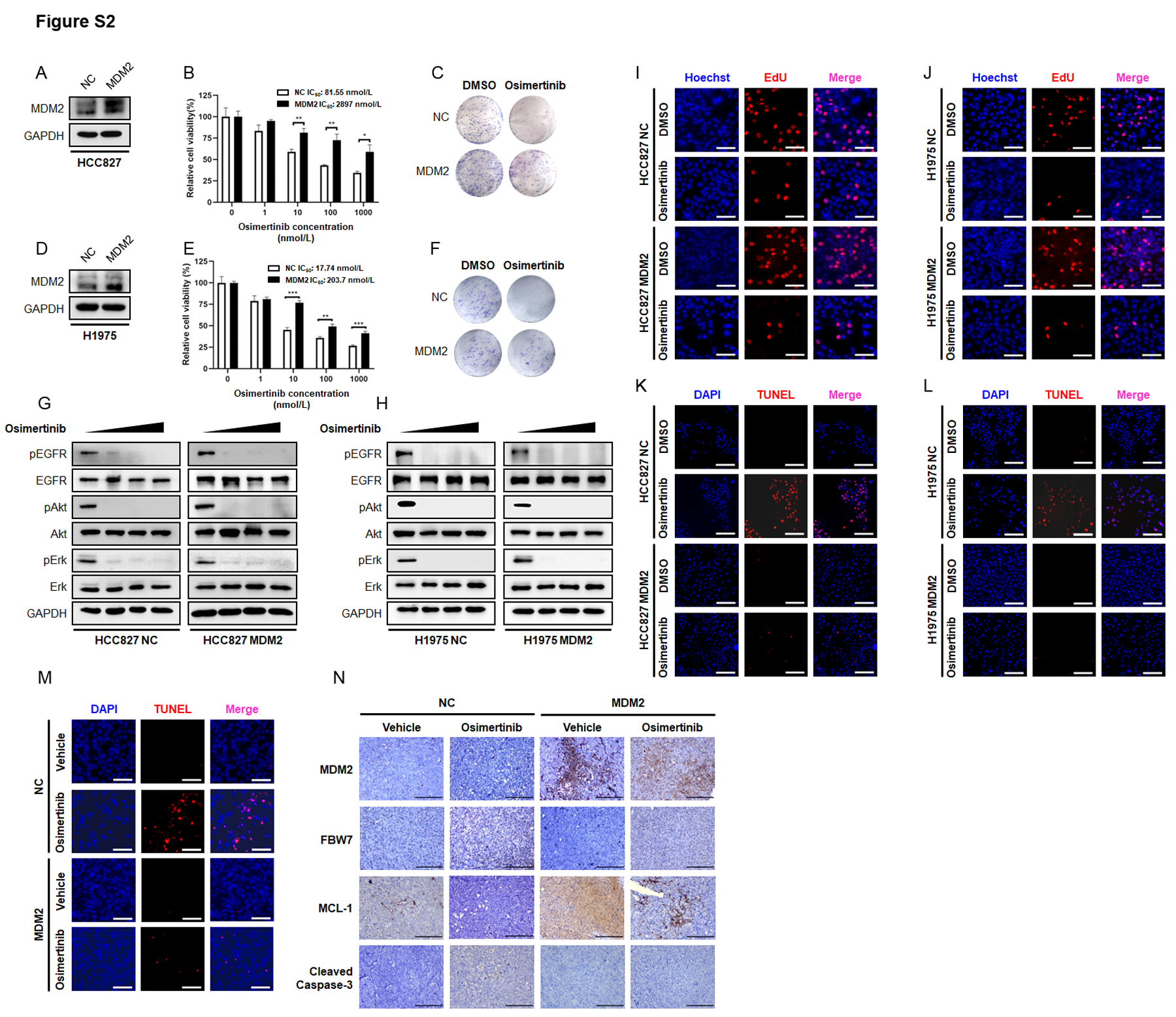

Supplement: Supplementary file 7 — Supplementary Material 7 [file 13046_2024_3220_MOESM7_ESM.tif]

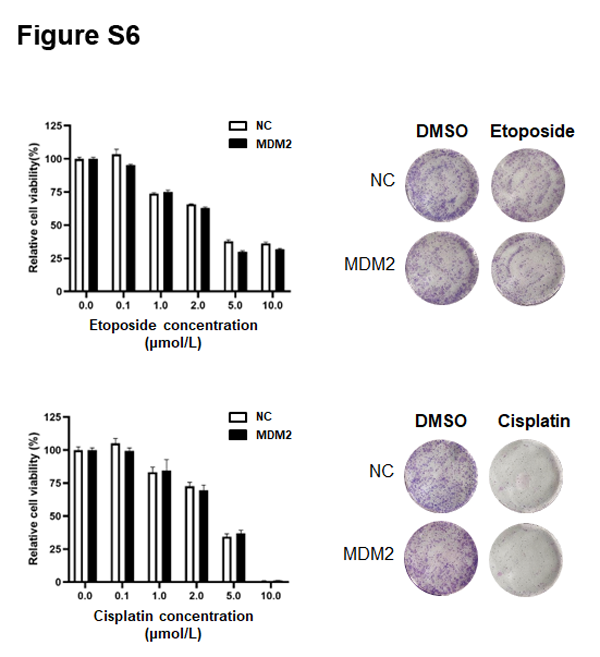

Supplement: Supplementary file 8 — Supplementary Material 8 [file 13046_2024_3220_MOESM8_ESM.tif]
