# Supplementary material for: MDM2 drives resistance to Osimertinib by contextually disrupting FBW7-mediated destruction of MCL-1 protein in EGFR mutant NSCLC
Source: J Exp Clin Cancer Res. 2024 Nov 15;43:302. doi: 10.1186/s13046-024-03220-7 (PMC11566350; doi:10.1186/s13046-024-03220-7)
Supplement: Supplementary file 5 — Supplementary Material 5 [file 13046_2024_3220_MOESM5_ESM.docx]

**Supplementary Table S1. Antibody sources for Western blot, IP and IHC.**

| **Antibody for Western blot** | **Brand & Catalog No.** | **Dilution** |
| --- | --- | --- |
| MDM2 | Abcam, ab259265 | 1/1000 |
| p-EGFR | Cell Signaling Technology, #3777 | 1/1000 |
| EGFR | Cell Signaling Technology, #4267 | 1/1000 |
| p-Akt | Cell Signaling Technology, #4060 | 1/1000 |
| Akt | Cell Signaling Technology, #9272 | 1/1000 |
| p-Erk | Cell Signaling Technology, #4370 | 1/2000 |
| Erk | Cell Signaling Technology, #4695 | 1/2000 |
| GAPDH | Proteintech, 60004-1-Ig | 1/20000 |
| β-actin | Proteintech, 60008-1-Ig | 1/5000 |
| MCL-1 | Cell Signaling Technology, #94296 | 1/1000 |
| BCL-2 | Cell Signaling Technology, #15071 | 1/1000 |
| BCL-xL | Cell Signaling Technology, #2764 | 1/1000 |
| BAX | Cell Signaling Technology, #5023 | 1/1000 |
| BIM | Cell Signaling Technology, #2933 | 1/1000 |
| PARP | Cell Signaling Technology, #9532 | 1/1000 |
| Cleaved Caspase-3 | Cell Signaling Technology, #9661 | 1/1000 |
| Cyclin E | Cell Signaling Technology, #20808 | 1/1000 |
| c-Myc | Cell Signaling Technology, #18583 | 1/1000 |
| FBW7 | Proteintech, 28424-1-AP | 1/1000 |
| p53 | Abcam, ab32389 | 1/1000 |
| Ub | R&D, MAB701 | 1/1000 |
| Myc tag | Abbkine, #A02060 | 1/2000 |
| Flag tag | Abbkine, #A02010 | 1/2000 |
| His tag | Abbkine, #A02050 | 1/2000 |
| HA tag | Abbkine, #A02040 | 1/2000 |
| HRP-Rabbit IgG | Cell Signaling Technology, #7074 | 1/5000 |
| HRP-Mouse IgG | Cell Signaling Technology, #7076 | 1/5000 |
|  |  |  |
| **Antibody for IP** | **Brand & Catalog No.** | **Dilution** |
| Flag tag | Sigma, F3165 | 1/500 |
| HA tag | Abbkine, #A02040 | 1/500 |
| Myc tag  FBW7  MDM2 | Abbkine, #A02060  Santa Cruz, sc-33196  Santa Cruz, sc-965 | 1/500  1/50  1/50 |
|  |  |  |
| **Antibody for IHC** | **Brand & Catalog No.** | **Dilution** |
| FBW7 | Santa Cruz, sc-33196 | 1/50 |
| MDM2 | Santa Cruz, sc-965 | 1/50 |
| MCL-1 | Abcam, ab32087 | 1/200 |
| Cleaved Caspase-3 | Cell Signaling Technology, #9661 | 1/400 |
