# Supplementary material for: MDM2 drives resistance to Osimertinib by contextually disrupting FBW7-mediated destruction of MCL-1 protein in EGFR mutant NSCLC
Source: J Exp Clin Cancer Res. 2024 Nov 15;43:302. doi: 10.1186/s13046-024-03220-7 (PMC11566350; doi:10.1186/s13046-024-03220-7)
Supplement: Supplementary file 6 — Supplementary Material 6 [file 13046_2024_3220_MOESM6_ESM.docx]

**Supplementary Table S2. siRNA target sequences.**

| p53 | 5′-AAUAUUCUCCAUCCAGUGGTT-3′ |
| --- | --- |
| MDM2 | 5′-AAGCTTGGCACGCCAAACAAA-3′ |
